# Differential Scaling of Synaptic Molecules within Functional Zones of an Excitatory Synapse during Homeostatic Plasticity

**DOI:** 10.1523/ENEURO.0407-19.2020

**Published:** 2020-04-07

**Authors:** Sridevi Venkatesan, Sandhya Subramaniam, Premchand Rajeev, Yukti Chopra, Mini Jose, Deepak Nair

**Affiliations:** Centre for Neuroscience, Indian Institute of Science, Bangalore, 560012, Karnataka, India

**Keywords:** homeostatic plasticity, immunocytochemistry, molecular organization of synapse, multiplicative scaling, super-resolution imaging, synaptic transmission and plasticity

## Abstract

Homeostatic scaling is a form of synaptic plasticity where individual synapses scale their strengths to compensate for global suppression or elevation of neuronal activity. This process can be studied by measuring miniature EPSP (mEPSP) amplitudes and frequencies following the regulation of activity in neuronal cultures. Here, we demonstrate a quantitative approach to characterize multiplicative synaptic scaling using immunolabelling of hippocampal neuronal cultures treated with tetrodotoxin (TTX) or bicuculline to extract scaling factors for various synaptic proteins. This approach allowed us to directly examine the scaling of presynaptic and postsynaptic scaffolding molecules along with neurotransmitter receptors in primary cultures from mouse and rat hippocampal neurons. We show robust multiplicative scaling of synaptic scaffolding molecules namely, Shank2, PSD95, Bassoon, and AMPA receptor subunits and quantify their scaling factors. We use super-resolution microscopy to calculate scaling factors of surface expressed GluA2 within functional zones of the synapse and show that there is differential and correlated scaling of GluA2 levels within the spine, the postsynaptic density (PSD), and the perisynaptic regions. Our method opens a novel paradigm to quantify relative molecular changes of synaptic proteins within distinct subsynaptic compartments from a large number of synapses in response to alteration of neuronal activity, providing anatomic insights into the intricacies of variability in strength of individual synapses.

## Significance Statement

Here we demonstrate a novel quantitative method based on rank ordered analysis to characterize multiplicative synaptic scaling from immunolabelling of hippocampal neuronal cultures after activity blockade. We show that along with glutamatergic receptors, several scaffolding molecules are scaled after blocking activity. This analysis paradigm can be generalized to any protein which alters its content post plasticity protocols. With the aid of conventional and super-resolution microscopy, we examine pools of AMPA type glutamatergic receptors, and confirm that they are scaled differentially within functional zones of synapses. Furthermore, we show that the AMPA receptor content within the postsynaptic density (PSD) and perisynaptic compartments are altered differentially during homeostatic scaling, indicating a differential regulation of receptors within various subsynaptic compartments during homeostatic plasticity.

## Introduction

Specific changes in synaptic activity are associated with cognitive processes such as learning and memory (Martin et al., 2000; Lamprecht and LeDoux, 2004). These changes alter both the molecular and morphologic characteristics of synapses and are referred to as synaptic plasticity. Last three decades of research have shown that the instantaneous distribution of synaptic molecules contributes to both the structure and function of individual synapses (Kandel et al., 2014; Bailey et al., 2015; Sossin, 2018). The spatiotemporal heterogeneity of AMPA type glutamatergic receptors at excitatory synapses have been directly correlated to short term plasticity and activity dependent changes in the synaptic strength (Henley and Wilkinson, 2016; Chen et al., 2018; Humeau and Choquet, 2019). Quantifying the variability in electrophysiological properties of AMPA receptor responses in synapses along with their localization and trafficking have enabled us to understand finer aspects of both basal synaptic transmission and activity dependent changes associated with it (Greger et al., 2017; Diering and Huganir, 2018). Homeostatic synaptic scaling is a form of synaptic plasticity where the excitatory synapses scale their strengths in response to prolonged alteration of neuronal activity. Homeostatic synaptic scaling was first experimentally demonstrated by suppressing neuronal activity in cultured cortical neurons with tetrodotoxin (TTX) for 48 h, which resulted in an increase in the amplitudes of miniature EPSCs (mEPSCs; Turrigiano et al., 1998). Subsequently, it has been demonstrated that this increase in mEPSC amplitudes is mediated by increased accumulation of postsynaptic AMPA receptors (O’Brien et al., 1998; Wierenga et al., 2005). Homeostatic scaling in response to activity suppression has been shown to involve structural and functional changes at both the presynaptic and postsynaptic compartments. These changes include modulation of the structure and size of the readily releasable pool, spine volume and postsynaptic density (PSD), as well as increased mEPSC frequency and/or amplitude (Turrigiano, 2012; Vitureira et al., 2012; Fernandes and Carvalho, 2016). Homeostatic scaling is also dependent on the maturation of neurons *in vitro*, with older neurons preferentially modulating the postsynaptic compartment (Wierenga et al., 2006; Han and Stevens, 2009). Homeostatic scaling down of excitatory synapses has also been observed on elevation of neuronal activity induced by bicuculline; competitive antagonist for GABA_A_ receptors at inhibitory synapses (Ibata et al., 2008; Tatavarty et al., 2013).

Homeostatic scaling is one of the major phenomena widely employed to understand synaptic plasticity. Our understanding of functional changes in synaptic activity has primarily evolved by correlating changes in the amplitude and frequency of mEPSCs (Han and Stevens, 2009; Kim et al., 2012; Turrigiano, 2012). These changes are often correlated with microscopic observation of changes in synaptic morphology and distribution of neurotransmitter receptor subunits in synapses. One of the most robust approaches to understand the variability in the properties of individual synapses using homeostatic scaling relies on comparing the mEPSC recordings between the control neurons and those that have undergone homeostatic scaling. This analysis differentiates the increase or decrease in global strength of the synapses along with their relative scaling factors for each condition (Kim et al., 2012). However, examining mEPSCs to study homeostatic scaling provides only a preview of all active synapses on the cell at any given time, while the variability underlying the structural and molecular changes in the presynaptic and postsynaptic compartments are overlooked. Although conventional microscopy data has shed light on instantaneous distribution and recycling of receptors in the synapses, it seldom focuses on the properties of individual synapses. Furthermore, advances in high-resolution microscopy in the recent years have enabled researchers to examine variability within individual synapses and map the topology of AMPA receptor distribution within functional zones such as the PSD and the perisynaptic region of excitatory synapses, spatially separated by few 100 nm (Chen et al., 2018; Scheefhals and MacGillavry, 2018; Humeau and Choquet, 2019). Here, we have studied the spatial heterogeneity of proteins at synapses using conventional microscopy and calculated their individual scaling factors with a method similar to the one used for electrophysiological recordings (Kim et al., 2012). We have extracted multiplicative scaling factors for several key presynaptic and postsynaptic scaffolding molecules like Shank2, PSD95, and Bassoon as well as AMPA receptor subunits, from primary neuronal cultures of both mice and rats after the induction of homeostatic scaling by activity suppression (Brandstätter et al., 2004; Schoch and Gundelfinger, 2006; Sheng and Hoogenraad, 2007; Chua et al., 2010; Sudhof, 2012). Likewise, we demonstrate that the same method can also be used to estimate scaling factors following synaptic scaling down induced by blockade of GABA_A_ receptors by bicuculline. Furthermore, with the aid of super-resolution microscopy of immunolabelled neurons, we were able to extract the population of AMPA receptors in both the postsynaptic and perisynaptic compartments. This enabled us to calculate the scaling factors within spatially discrete functional zones of synapses segregated by few 100 nm. Our study provides an easily adaptable approach to obtain anatomic insights into the molecular basis of homeostatic changes across multiple synapses.

## Materials and Methods

### Neuronal cultures

Hippocampal neurons from postnatal day 1 (P1) C57B mouse pups and postnatal day 0–1 Sprague Dawley rat pups were cultured on poly-L-lysine (Sigma) coated glass coverslips following the protocol as reported previously (Beaudoin et al., 2012). The dissected hippocampi were briefly trypsinized (Thermo Fisher Scientific) and dissociated in Hibernate-A (Thermo Fisher Scientific) media supplemented with 0.5× B27 (Thermo Fisher Scientific), 0.25× GlutaMAX (Thermo Fisher Scientific), and 100 μg/ml Normocin (Invivogen). The dissociated neurons were resuspended in Neurobasal A (Thermo Fisher Scientific) medium supplemented with 0.5× B27, 0.25× GlutaMAX and 100 μg/ml Normocin (InvivoGen) and plated on poly-L-lysine coated coverslips. Fresh Neurobasal A medium was supplemented every week to maintain the culture. All experiments involving animals were performed in accordance with institutional guidelines for the use and care of animals under the approval of the animal ethics committee.

### Homeostatic plasticity induction protocol

Induction of homeostatic scaling in pyramidal neurons was conducted by incubation of days in vitro (DIV) (unless otherwise specified) mouse and rat hippocampal neuronal cultures with 2 μM TTX (Alamone labs) for 24–48 h as specified or 40 μM bicuculline (Tocris Bioscience) for 48 h. The cultures treated with TTX will be referred to as TTX dataset and those treated with bicuculline will be referred to as bicuculline dataset. Untreated cultures of the corresponding age were used as control.

### Live surface labeling of AMPA receptors

For surface labeling of AMPA receptors, the neuronal cultures were incubated in conditioned Neurobasal media containing 25.5 μg/ml GluA2- N-terminal antibody for 10 min on ice (Sobolevsky et al., 2009; Nair et al., 2013).

### Immunocytochemistry

The neuronal cultures were fixed in 4% paraformaldehyde and 4% sucrose in PBS for 10 min at 4°C, permeabilized with 0.25% Triton X-100 and blocked with 10% BSA solution for 30 min. Primary and secondary antibodies were diluted in 3% BSA and incubated for 1 h and 45 min at room temperature, respectively. Coverslips were mounted on glass slides with ProLong Diamond antifade reagent (Thermo Fisher Scientific).

The primary antibodies used in this study were, guinea pig anti-Shank2 (Synaptic Systems, catalog no. 162204), mouse anti-Bassoon (Synaptic Systems, catalog no. 141021), rabbit anti-GluA1 (Synaptic Systems, catalog no. 182003), guinea pig anti-GluA2 (Synaptic Systems, catalog no. 182105), mouse anti-PSD95 (Invitrogen, catalog no. MA1-046), and guinea pig anti-Bassoon (Synaptic Systems, catalog no. 141004). The above-mentioned antibodies were used at a dilution of 1:500. Antibody against the extracellular domain of the GluA2 subunit of AMPA receptor was a kind gift from Eric Gouaux, Vollum Institute, and it was used at a dilution of 1:200. The secondary antibodies used were, goat anti-mouse STAR RED [Abberior, item no. STRED; for stimulated emission depletion (STED) microscopy], goat anti-guinea pig Alexa Fluor 647 (Life Technologies, catalog no. A21450), goat anti-guinea pig Alexa Fluor 594 (Life Technologies, catalog no. A11076), goat anti-mouse Alexa Fluor 568 (Life Technologies, catalog no. A11004), goat anti-rabbit Alexa Fluor 532 (Life Technologies, catalog no. A11009), and goat anti-mouse Alexa Fluor 488 (Life Technologies, catalog no. A11029). The secondary antibodies were used at a dilution of 1:500.

### Confocal microscopy

Images were acquired using a Zeiss confocal laser scanning microscope (Zeiss LSM 780 and Zeiss LSM 880, Carl Zeiss) equipped with a 63× oil immersion objective lens of numerical aperture 1.4. The refractive index of oil was 1.51. The sampling size of each image acquired on the Zeiss microscope was 90 nm per pixel for the LSM 780 and 44 nm per pixel for the LSM 880, while using a zoom of 3. Sections of 380 nm along the *z*-axis were imaged with an axial sampling of 30–70 nm. For tile scan imaging to differentiate proximal and distal synapses, a single plane tile scan was taken with the help of a confocal microscope (Zeiss LSM 880) keeping the cell body at the center and sampling at 190 nm to cover a total area of 270 × 270 μm^2^

### STED microscopy

STED images were acquired with a sampling of 15 nm per pixel using a 100× oil immersion objective mounted on an Abberior STED microscope (Abberior GmbH). STED laser of 770 nm was used and the imaging regions were chosen randomly. Optical sectioning was performed similar to the conventional confocal microscope, as described in the previous section.

### Image analysis

Image analysis was done using MetaMorph software (MetaMorph 7.8, Molecular Devices). Maximum projections of the Z-stacks acquired using the Zeiss confocal microscope were used for analysis. Synaptic protein puncta were identified using adaptive thresholding on each channel. The same adaptive threshold was used for the control and treated dataset. By thresholding, fluorescent puncta with an area above 0.02 and 0.038 μm^2^ were selected for mouse and rat neurons, respectively, for the confocal images. Fluorescent puncta with an area of 0.02–0.54 μm^2^ (PSD area estimated in Harris and Stevens, 1989) were chosen for the STED images. The properties of the puncta such as area, average, and total fluorescence intensity were measured using Integrated morphometry analysis. A minimum length filter of 0.18 μm was used in Integrated morphometry analysis.

We quantified the percentage of active synapses in the putative synaptic masks that we obtained from neuronal processes using diffraction limited microscopy, after segmentation with the aid of two additional paradigms. In the first paradigm, we performed a co-labeling of the postsynaptic marker of interest (Shank2) with a presynaptic marker (Bassoon). We then performed segmentation according to the previously mentioned measurement criteria and binarized the regions corresponding to both the presynaptic and postsynaptic markers. We measured whether any binarized signal could be detected in the segmented image of the presynaptic marker corresponding to a region where the postsynaptic compartment was identified. Regions recording a signal would be considered as a functional synapse by the presence of both presynaptic and postsynaptic machinery. In the second paradigm, we co-labeled two postsynaptic markers (PSD95 and Shank2) and repeated the protocol to identify the presence of these markers together, indicating functional postsynaptic machinery. We found that in 95.5% (*n* = 10 cells) of synapses, there was an overlap between presynaptic and postsynaptic compartments and in 99.9% (*n* = 10 cells) of synapses, there was an overlap between the two postsynaptic markers. These results further confirmed that the putative PSD masks identified by our segmentation protocol detect functional synapses with a high degree of accuracy. Thus, we will refer to the molecules of interest (subunits of AMPA receptors and scaffolding molecules) detected within these masks as “synaptic” in the rest of the study.

To study proximal and distal synapses, square regions of size 33 × 33 μm were chosen within 66 μm of the cell body and above 132 μm from the cell body and image analysis was performed with the help of adaptive thresholding and integrated morphometry analysis.

### Statistics

The normality of the distributions was checked by D’Agostino–Pearson omnibus normality test. The frequency distributions used for constructing the probability density function histograms and the cumulative probability distribution function curves were normalized. Two-sample Kolmogorov–Smirnov (KS) test was used to compare the distributions of control, treated and scaled data. For the KS test, significance level was set to 0.001 due to the high sample size. The *p* value was differentiated into stars (*) by the following benchmarks: *10^−3^ ≥ *p* ≥ 10^−4^; **10^−4^ ≥ *p* ≥ 10^−5^; ***10^−5^ ≥ *p* ≥ 10^−6^; ****p < 10^−6^. Aforementioned definition of benchmarking of *p* values is consistent with results and figures unless otherwise stated. Unpaired *t* test was used to compare cell-wise averages of average intensity between control and treated data, with 0.05 as the significance level.

To generate rank-ordered plots of treated versus control fluorescence intensities, data of all puncta from all cells were pooled for the control and treated conditions. The same number of puncta were chosen randomly from both control and treated data. These data were rank ordered from lowest to highest fluorescence intensity and the treated data were plotted against the control data and a linear fit was calculated. The slope of the linear fit gave an estimate for the multiplicative scaling factor. To determine the accurate scaling factor using the method used in Kim et al. (2012), the treated distribution was scaled down or up by an arbitrary multiplicative factor and only the scaled values greater than a threshold (the minimum intensity in the control condition) were included, i.e., scaled data = (treated data/scaling factor) > threshold. This was done for a range of hypothetical scaling factors and the KS test was used to compare the scaled distribution with the control distribution. The scaling factor corresponding to the highest *p* value was considered as the most accurate. This process was repeated 100 times from different samplings of the puncta to obtain the average scaling factor ± SEM.

Additionally, the control and scaled data were also compared with the help of the Anderson–Darling (AD) test to determine whether there would be a different result with respect to the KS test (Scholz and Stephens, 1987; Engmann and Cousineau, 2011). The AD test gives us a set of critical values (i.e., 0.325, 1.226, 1.961, 2.718, 3.752) for different confidence intervals (i.e., 75%, 90%, 95%, 97.5%, and 99%, respectively). A statistic value is generated for the compared data which must be compared against the critical values to determine significance. The statistic value must be lower than the critical value for the null hypothesis (i.e., the two distributions are not significantly different) to hold true. We only report results of the AD test if they were different from the KS test.

## Results

### Multiplicative scaling of synaptic proteins determined using rank order analysis

**Figure 1.**
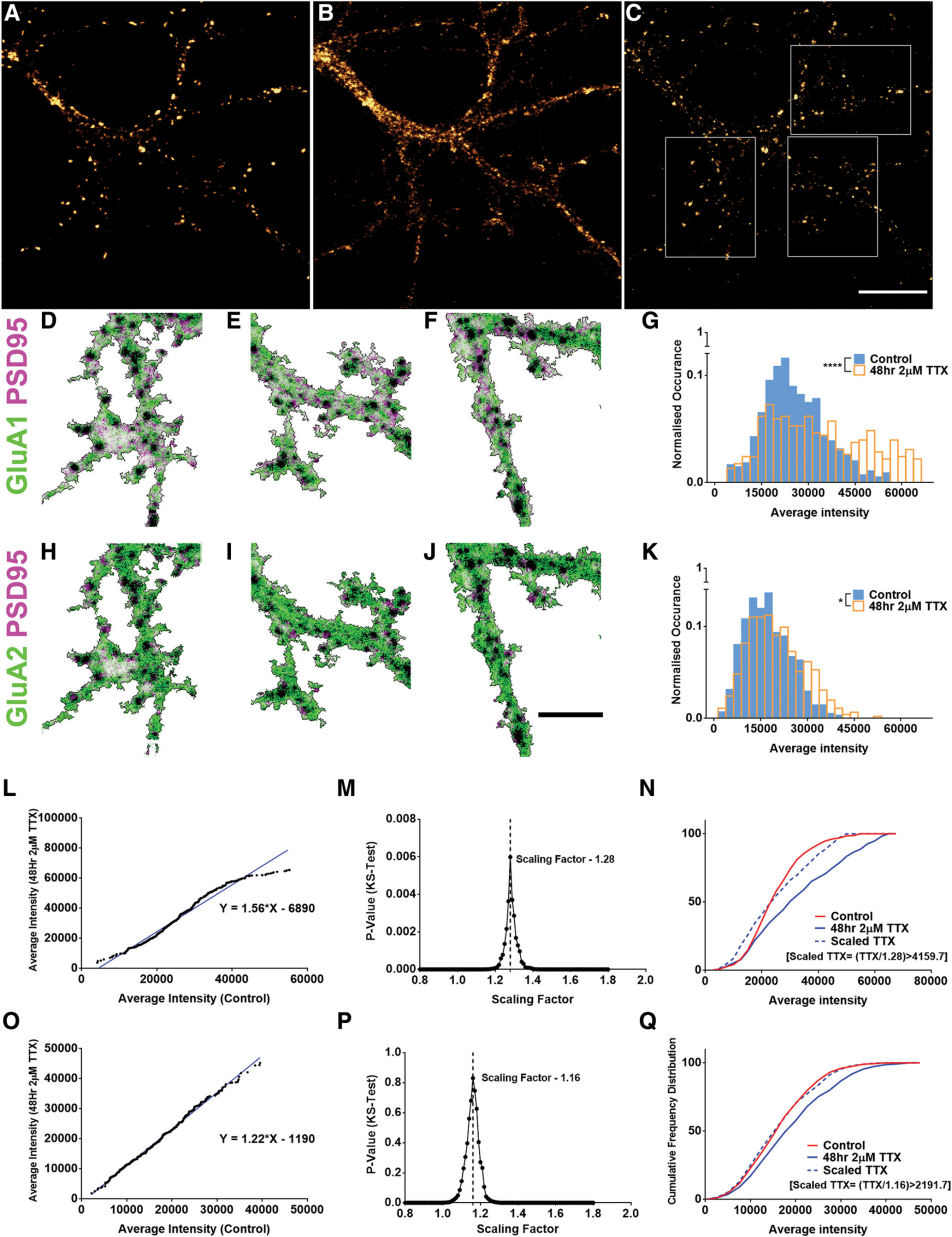
Multiplicative scaling of excitatory synapses using rank order analysis. ***A–C***, Mice hippocampal neurons of DIV14 treated for 48 h with 2 μM TTX to study homeostatic synaptic scaling. The cultures were fixed and immunolabelled for the C terminus of AMPA receptor subunits GluA1 (***A***), GluA2 (***B***), and a PSD protein PSD95 (***C***). ***D–F***, ***H–J***, Representative dendritic compartments of the images in ***A–C*** with PSD95 in magenta and GluA1 (***D–F***) or GluA2 (***H–J***) in green. Scale bars: 10 μm (***A–C***) and 5 μm (***D–F***, ***H–J***). ***G***, ***K***, A histogram was plotted for the average intensity of GluA1 (***G***) or GluA2 (***K***) per PSD95 puncta between the control and the TTX-treated conditions (*n* = 534 and 454 synapses for control and TTX dataset, respectively, *p* < 10^−6^ for GluA1 and *p* < 10^−3^ for GluA2, respectively, KS test). ***L***, ***O***, A random sample of 400 intensity values were chosen, rank ordered and plotted to provide a scaling equation for GluA1 (***L***; y = 1.56x – 6890) and GluA2 (***O***; y = 1.22x − 1190). ***M***, ***P***, Using the slope of the equation as a reference, the TTX dataset was scaled using multiple values and compared with the control. The scaling factor providing the maximum *p* value between the scaled-TTX and control dataset was chosen as the multiplicative scaling factor for GluA1 (1.28, *p* = 0.006, KS test; ***M***) and GluA2 (1.16, *p* = 0.80, KS test; ***P***). ***N***, ***Q***, Cumulative frequency distribution between control, TTX, and scaled-TTX for GluA1 (***N***) and GluA2 (***Q***) showed no significant difference between the control and scaled-TTX distributions.

Homeostatic synaptic scaling is accompanied by increased expression of GluA1 and GluA2 AMPA receptor subunits. In order to study homeostatic synaptic scaling, we treated primary hippocampal neurons of DIV14 from P1 C57B mice with 2 μM TTX for 48 h. We performed immunocytochemistry for AMPA receptor subunits, GluA1 and GluA2, and for the postsynaptic scaffolding molecule PSD95 (Fig. 1*A–C*) to study the effect of homeostatic plasticity on excitatory postsynaptic proteins. Confocal imaging was performed on a section of dendrite of pyramidal neurons identified by morphology. The GluA1 and GluA2 levels measured within PSD95 puncta are representative of synaptic AMPAR levels, and thereby in direct correlation with the electrophysiological characteristics encoded by mEPSCs. The overlay of GluA1 and GluA2 with PSD95 are shown in Figure 1*D–F*,*H–J*, respectively. We pooled the average fluorescence intensities of GluA1 and GluA2 segmented within the PSD95 puncta, representing the variability of PSD sizes, across all cells in control and TTX-treated conditions to generate frequency distribution histograms (*n* = 9 and 12 cells, 534 and 454 synapses for control and TTX dataset, respectively; Fig. 1*G*,*K*). Comparing the intensity histograms between the control and TTX dataset showed a significant increase in the synaptic levels of both GluA1 and GluA2 subunits of AMPA receptors (synaptic GluA1: *p* < 10^−6^ and synaptic GluA2: *p* < 10^−3^, two-sample KS test) after induction of homeostatic plasticity.

To determine whether the nature of this scaling was multiplicative and to compute the exact multiplicative scaling factor, we performed rank order analysis on the average puncta intensities of control and TTX datasets. A total of 400 puncta were picked randomly from both the control and TTX datasets for synaptic GluA1 and GluA2. Their intensities were arranged in ascending order and the TTX dataset was plotted against the control (Fig. 1*L*,*O*). The data fit to a linear model for both synaptic GluA1 (Fig. 1*L*; linear fit: y= 1.56× – 6890, goodness of fit *R*^2^ = 0.97) and GluA2 (Fig. 1*O*; linear fit: y = 1.22x − 1190, *R*^2^ = 1). The slope of the linear fit could be considered as the multiplicative factor, however, the presence of an intercept value in the linear fit indicated an additive component to the synaptic scaling.

The measurement of mEPSCs from primary cultured neurons has a set detection threshold for both control and TTX-treated conditions. In the rank-ordered plot, the lowest value of the TTX dataset should correspond to the lowest value in the control dataset. However, the lowest value of the TTX-treated dataset seemed to be offset from that of the control value due to the detection threshold, contributing to the additive component of the scaling equation as discussed previously (Kim et al., 2012). Therefore, a true multiplicative scaling factor could be calculated only for data where no thresholds have been applied. To account for this thresholding error and to determine the multiplicative scaling factor, we used the method previously reported to characterize scaling of mEPSC amplitudes (Kim et al., 2012). Here, the scaled-TTX distribution was obtained by dividing the TTX values by arbitrary scaling factors, and only values greater than the threshold (lowest value in control) were included in the scaled-TTX distribution. The scaling factor that provided the highest *p* value on comparing scaled-TTX and control distribution was taken as the multiplicative scaling factor. Figure 1*M*,*P* plots the *p* values from KS tests comparing the control and scaled-TTX distributions obtained with a range of scaling factors. The highest *p* value corresponded to a scaling factor of 1.28 for synaptic GluA1, indicating that 1.28 was the multiplicative scaling factor for synaptic GluA1, whereas, for synaptic GluA2, it was 1.16. Figure 1*N*,*Q* shows that the scaled-TTX distribution obtained by dividing the TTX dataset with the above mentioned scaling factors was not significantly different from the control distribution for synaptic GluA1 (*p* = 0.006, KS test) or GluA2 (*p* = 0.80, KS test). The sampling of the dataset was repeated 100 times and the average multiplicative scaling factors for synaptic GluA1 and GluA2 were determined to be 1.30 ± 0.002 and 1.16 ± 0.001, respectively. Thus, a multiplicative scaling factor was derived using this method on microscopy dataset, a task previously achieved only by electrophysiological characterization of mEPSC recordings.

We used the AD test to compare the TTX and scaled-TTX distributions. We observed that except for the GluA1 data, the AD test provided a statistic value far below the critical values for GluA2, confirming that the scaled-TTX distributions were not significantly different from the control distributions. For GluA1, the statistic value for control versus scaled-TTX was found to be greater than the critical value, but less than that of the versus TTX dataset. We used the AD test to calculate the multiplicative scaling factor by determining the scaling factor at which the test statistic for control versus scaled-TTX showed the smallest value for GluA1 and GluA2, and we obtained a scaling factor similar to the one obtained with the help of the KS test indicating that the scaling factor is indeed comparable across the statistical tests

Additionally, we compared the scaling factor derived from a mean value per cell analysis which is a commonly used method, to that derived from the above described method which involves random sampling of synapses. We examined the cell-wise means of average fluorescence intensity of GluA1 puncta shown in Figure 1*A* and observed that the mean of GluA1 average intensity of TTX-treated cells showed significant scaling in comparison to that of the control cells, corresponding to a scaling of 1.12 ± 0.028 (control – 1.00 ± 0.027, *t*_(19)_ = 2.94, *p* = 0.008, unpaired *t* test). We observed that this scaling factor estimated from cell-wise averages is lesser than the one obtained from the random sampling method, i.e., 1.30 ± 0.002. The cell-wise average value provides equal weightage for every synapse for a cell without taking synaptic heterogeneity into account. Thus, cell-wise average maybe useful to detect the presence of scaling as an overall effect, but it is not reflective of true multiplicative changes within synapses.

### Multiplicative scaling of scaffolding molecules during homeostatic scaling in rodents

**Figure 2.**
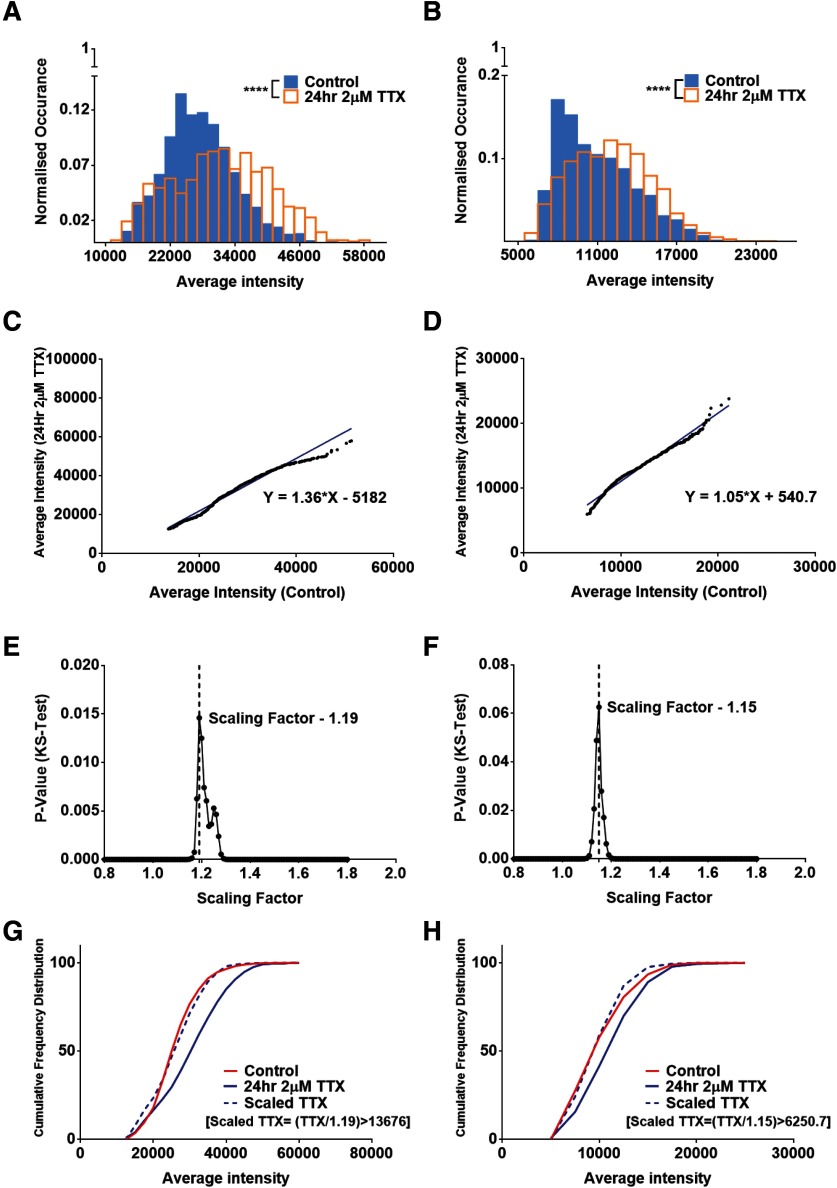
Multiplicative scaling of synaptic scaffolding molecules during homeostatic scaling in rodents. ***A***, ***B***, Histogram comparing the average intensity of Shank2 per puncta in the control and TTX conditions showed positive scaling after 24 h of treatment for both mouse (***A***) and rat (***B***) cultures (*n* = 1889 and 826 synapses for mouse and 1592 and 1821 synapses for rat, for control and TTX dataset, respectively, *p* < 10^−6^, KS test). ***C***, ***D***, A total of 800 random average intensity data points were chosen, rank ordered, and plotted to provide a scaling equation for mouse (***C***; y = 1.36x – 5182) and rat (***D***; y =1.05x + 540.7) cultures. ***E***, ***F***, The scaling factor providing the maximum *p* value between control and scaled-TTX datasets was chosen as the multiplicative scaling factor for both mouse (***E***; 1.19, *p* = 0.015, KS test) and rat (***F***; 1.15, *p* = 0.063, KS test) cultures. ***G***, ***H***, A cumulative frequency distribution was plotted for control, TTX, and the scaled-TTX (calculated with the help of the scaling factor) datasets for the mouse (***G***) and rat (***H***) showing no significant difference between the control and the scaled-TTX dataset. For comparison of multiplicative scaling of other synaptic scaffolding molecules, please refer to Extended Data Figure 2-1. A detailed analysis of sampling efficiency for calculating multiplicative scaling factor and significance of scaling factors are detailed in Extended Data Figures 2-2, 2-3, respectively. Analysis referring to accuracy of scaling in homeostatic plasticity is shown in Extended Data Figure 2-4.

We have observed rank order analysis to be an efficient tool to demonstrate multiplicative scaling and to calculate an accurate scaling factor for postsynaptic receptor molecules. In order to test the robustness of this method to study multiplicative scaling across rodent species, we studied postsynaptic scaffolding molecules in primary mice and rat cultures. Primary hippocampal cultures of DIV14 from P0 C57B mouse and P0/P1 Sprague Dawley rat pups were treated with 2 μM TTX for 24 h, and immunocytochemistry was performed for the PSD protein Shank2. Figure 2*A* shows the distribution of fluorescence intensities for Shank2 puncta in the control and TTX-treated mouse neuronal cultures (*n* = 10 cells, 1889 and 826 synapses for control and TTX dataset, respectively), whereas Figure 2*B* shows the same for rat neuronal cultures (*n* = 10 cells, 1592 and 1821 synapses for control and TTX dataset, respectively). Figure 2*A*,*B* demonstrates a significant scaling of Shank2 in the TTX-treated cultures compared with control, as determined by a two-sample KS test (Fig. 2*A*,*B*; *p* < 10^−6^).

10.1523/ENEURO.0407-19.2020.f2-1 Extended Data Figure 2-1 Multiplicative scaling of scaffolding molecules in rodents. Immunocytochemistry was performed for PSD95 in mouse and rat cultures (DIV14) and Bassoon in mouse cultures (DIV21) following treatment with 2 μM TTX for 24 h. ***A–C***, Histograms constructed for the average intensity of PSD95 and Bassoon in both control and TTX-treated condition showed significant scaling in both mouse (***A***, ***C***) and rat (***B***) cultures [*n* = 1836 and 977 synapses for mouse PSD95, *p* < 10^−5^ (***A***), 1747 and 2072 synapses for rat PSD95, *p* < 10^−6^ (***B***) and 751 and 1798 synapses for mouse Bassoon, *p* < 10^−6^ (***C***), KS test]. ***D–F***, A total of 800 random puncta (700 in the case of mouse Bassoon) were chosen from both the control and TTX dataset and plotted to obtain the scaling equation for mouse (PSD95: y = 1.32x – 5175; Bassoon: y = 1.32x – 2573; ***D***, ***F***) and rat (PSD95: y = 1.30x – 1604; ***E***) data. ***G–I***, The TTX dataset was scaled using a number of arbitrary scaling factors and compared to the control. The factor providing the largest *p* value was chosen as the multiplicative scaling factor for mouse (PSD95 – 1.11, *p* = 0.026; Bassoon – 1.25, *p* = 0.14, KS test; ***G***, ***I***) and rat (1.2, *p* = 0.106, KS test; ***H***) cultures. ***J–L***, A cumulative frequency distribution was plotted for the control, TTX, and scaled-TTX showing that the control and the scaled-TTX dataset were not significantly different for either mouse (***J***, ***L***) or rat (***K***) cultures. Download Figure 2-1, EPS file.

10.1523/ENEURO.0407-19.2020.f2-2 Extended Data Figure 2-2 Sampling efficiency for calculating multiplicative scaling factor. ***A***, Rat hippocampal neurons (DIV14) were treated with 2 μM TTX for 48 h and immunolabelled for Shank2. The Shank2 confocal dataset for this condition was sampled at 200, 400, 600, 800, 1000, 1200, 1400, 1600, and 1800 data points for both the control and TTX datasets. A scaling factor was calculated 100 times for each sampling level with the help of previously described methods. The range of scaling factors obtained for each sampling level is plotted in the form of a box and whiskers plot (***A***). ***B***, A graph of the sampling factor variance was plotted against the sampling level for the dataset. Download Figure 2-2, EPS file.

**Figure 3.**
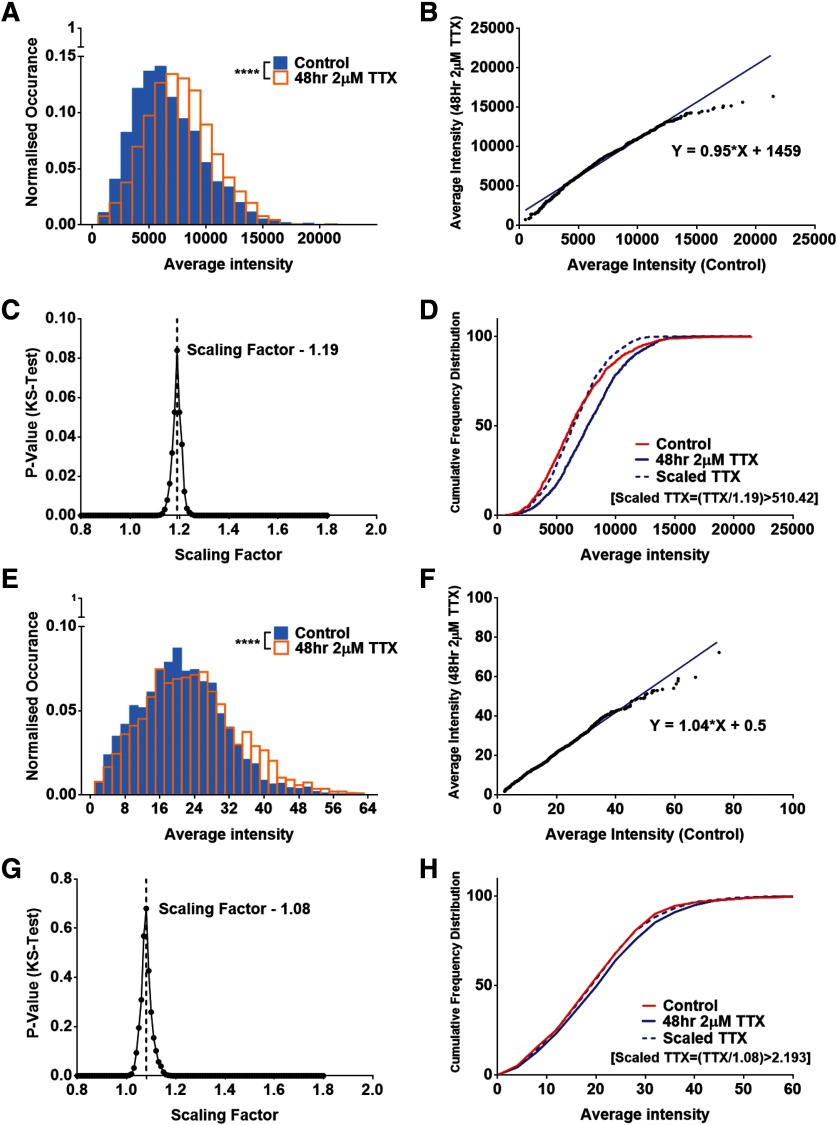
Differential scaling of synaptic AMPA receptor pools at excitatory synapses during homeostatic scaling. ***A***, ***E***, Histograms were constructed to compare the control and TTX dataset for the average intensity of GluA2 segmented within the Shank2 puncta for global synaptic GluA2 using the C-terminal antibody (***A***) and surface gluA2 using the N-terminal antibody (***E***); *n* = 1893 and 1781 synapses for global and 2259 and 2411 for surface GluA2, for control and TTX dataset, respectively, *p* < 10^−6^, KS test. ***B***, ***F***, A total of 1000 random puncta were chosen from both the control and TTX dataset, rank ordered, and plotted to provide the scaling equation for global gluA2 (***B***; y = 0.95x + 1459) and surface gluA2 (***F***; y = 1.04x + 0.5). ***C***, ***G***, The random sample of the TTX dataset was scaled down using multiple prospective scaling factors and the factor providing the maximum *p* value between the control and the scaled-TTX dataset was chosen as the multiplicative scaling factor for global gluA2 (***C***; 1.19, *p* = 0.084, KS test) and surface gluA2 (***G***; 1.08, *p* = 0.680, KS test). ***D***, ***H***, The cumulative distribution was plotted between the control, TTX, and the scaled-TTX (obtained with the help of the scaling factor) for the global (***D***) and surface (***H***) GluA2 levels, indicating no significant difference between the control and the scaled-TTX dataset. A gallery of immunocytochemical images for global and surface distribution of GluA2 is presented in Extended Data Figure 3-1.

10.1523/ENEURO.0407-19.2020.f2-3 Extended Data Figure 2-3 Significance of a low scaling factor. ***A–I***, The Shank2 confocal data for DIV14 rat cultures treated with 2 μM TTX for 48 h and sampled at 200, 400, 600, 800, 1000, 1200, 1400, 1600, and 1800 data points for both the control and TTX data. The sampled data from the control and TTX dataset were compared within themselves as well as between each other (i.e., control to control, TTX to TTX, and control to TTX); 100 scaling factors were obtained in each case. A cumulative frequency distribution was plotted for the scaling factors obtained in the case of control versus control, TTX versus TTX, and control versus TTX comparison for the sampling levels 200 (***A***), 400 (***B***), 600 (***C***), 800 (***D***), 1000 (***E***), 1200 (***F***), 1400 (***G***), 1600 (***H***), 1800 (***I***). ***J***, The scaling factors obtained for each sampling level was compared between the control versus control, TTX versus TTX, and control versus TTX dataset with the help of a KS test, and the *p* value was plotted against the sampling level. Download Figure 2-3, EPS file.

**Figure 4.**
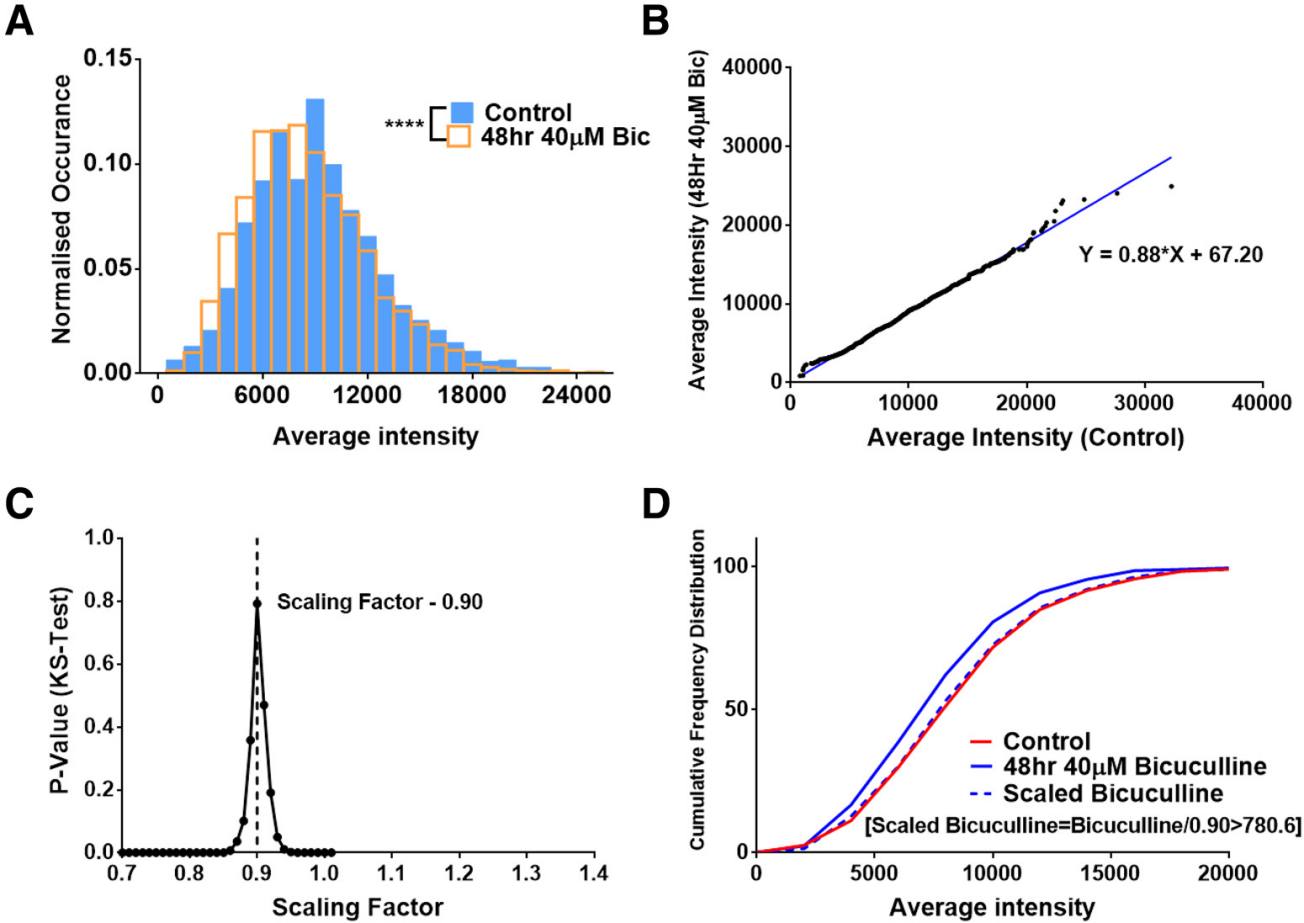
Scaling down of synaptic pool of surface AMPA receptors. ***A***, The normalized frequency distribution of average intensity of surface expressed synaptic GluA2 segmented within Shank2 puncta is plotted for control neurons and neurons treated with 40 μM bicuculline for 48 h. There is a a significant difference between the control and bicuculline dataset (*n* = 10 cells, 1697 and 2988 synapses for control and bicuculline dataset, respectively, *p* < 10^−6^). ***B***, A total of 1300 synapses were randomly sampled from the control and bicuculline dataset, rank ordered and plotted to obtain the scaling equation (y = 0.885x + 67.20). ***C***, The sampled bicuculline dataset was scaled with a number of arbitrary multiplicative scaling factors and compared with the control data. The scaling factor providing the maximum *p* value was chosen as the multiplicative scaling factor (0.90, *p* = 0.791). ***D***, The cumulative distribution of the control, bicuculline and scaled bicuculline population was plotted showing that the scaled bicuculline population was not significantly different from the control distribution.

10.1523/ENEURO.0407-19.2020.f2-4 Extended Data Figure 2-4 Accuracy of scaling in homeostatic plasticity of Shank2 at 48 h. ***A***, The maximum *p* values obtained while comparing 1000 randomly sampled synapses from the control and TTX distribution during the calculation of scaling factor repeated 500 times. ***B***, Gaussian fits of the normalized frequency distribution of scaling factors obtained while comparing the control and TTX datasets for each sampling size repeated 500 times. ***C***, Gaussian fit of the normalized frequency distribution of the error from mean of the scaling factors obtained in Figure 1*B*. ***D***, Tabular column detailing the average scaling factor and SD of error obtained for 1000 randomly sampled synapses from the control and TTX dataset repeated 100, 500, and 1000 times Download Figure 2-4, EPS file.

To measure the scaling factor, we performed rank order analysis on 800 Shank2 puncta randomly chosen from the control and TTX datasets for both the mouse and rat data. Figure 2*C*,*D* shows the linear fit to the plot of rank ordered TTX versus control intensities of Shank2. Similar to AMPA receptor subunits, the data fit to a linear model for both the mouse (Fig. 2*C*; linear fit: y = 1.36x – 5182, goodness of fit *R*^2^ = 0.97) and rat data (Fig. 2*D*; linear fit: y =1.05× + 540.7, *R*^2^ = 0.98), which supported multiplicative scaling. The scaling factor corresponding to the highest *p* value on comparing control and scaled-TTX distributions was 1.19 for the mouse culture (Fig. 2*E*) and 1.15 for the rat culture (Fig. 2*F*). These scaling factors were used to empirically scale down the TTX distribution and the cumulative frequency distributions of the control, TTX and scaled-TTX distributions are shown in Figure 2*G*,*H*. We observed that the scaled-TTX distribution was not significantly different from the control distribution for both the mouse (Fig. 2*G*; *p* = 0.015, KS test) and the rat data (Fig. 2*H*; *p* = 0.063, KS test). After repeating the sampling 100 times, we calculated a scaling factor of 1.19 ± 0.001 and 1.14 ± 0.001 for the mouse and rat Shank2 data, respectively. Thus, we were able to conclude that this quantitative method of analyzing immunocytochemical data to estimate scaling factors can be reproduced across species and synaptic proteins.

We also used the aforesaid method to determine multiplicative scaling factor following 24 h of TTX treatment in mouse and rat cultures for other scaffolding molecules. The scaling factor for PSD95 in primary neuronal cultures of mice was 1.1 (Extended Data Fig. 2-1*A*,*D*,*G*,*J* control), whereas it was 1.2 for rat (Extended Data Fig. 2-1*B*,*E*,*H*,*K*). The scaling factor for the presynaptic protein Bassoon in primary neuronal cultures of mice was 1.25 (Extended Data Fig. 2-1*C*,*F*,*I*,*L*). Along with the changes in average intensity, total intensity was also characterized as a tool to understand increase in total protein expression level after the induction of homeostatic plasticity. We measured scaling factor using the total puncta intensity of PSD95 in both mouse and rat hippocampal neurons after 48 h of TTX treatment. We obtained a scaling factor of 1.46 ± 0.005 and 1.39 ± 0.005 for the mouse and rat data, respectively, which was in agreement with previous observations (Sun and Turrigiano, 2011). We observed that the multiplicative scaling was comparable between Shank2 and PSD95 at synapses. Similar to PSD95, Shank2 is also known to associate with several molecules at the PSD (Brandstätter et al., 2004; Martinez-Monedero et al., 2016). Furthermore, Antibodies against Shank2 were available from a Guinea pig host which was more suitable in combination with several AMPA receptor subunit antibodies, which were of key interest to this paper. Therefore, we have used Shank2 as a postsynaptic marker for the rest of our study.

Thus, the proposed novel method could be used to determine precise multiplicative scaling factors for both presynaptic and postsynaptic proteins as well for any protein involved in homeostatic synaptic plasticity.

### Effect of sampling on the accuracy of measurement of the scaling factor

We estimated the effect of the sample size used on the accuracy of the scaling factor measured using this immunocytochemical method. In order to determine the accurate sample size to be accounted for calculating the scaling factor, we treated primary hippocampal cultures of DIV14 from P0/P1 Sprague Dawley rat pups with 2 μM TTX for 48 h, and immunocytochemistry was performed for Shank2. The data were sampled to various degrees, i.e., random samples of 200, 400, 600, 800, 1000, 1200, 1400, 1600, and 1800 data points were taken from the total dataset. The process was repeated 100 times to determine the mean scaling factor ± SD. The distribution of scaling factors for each sample size were compared (Extended Data Fig. 2-2*A*). The variance of the scaling factor estimate was plotted against the sample size. The inflection point of this curve lay close to 800, suggesting that a sample size of 800–1000 (40–50% of the total) puncta was sufficient to measure an accurate scaling factor (as indicated by the dashed lines in Extended Data Fig. 2-2*B*). In order to confirm that a low scaling factor indicated multiplicative scaling, similar sampling was done for the control and the TTX datasets individually and compared within each set, i.e., randomly chosen non-overlapping control datasets were compared with each other for all aforementioned sample sizes. The TTX dataset were also analyzed in a similar manner. Scaling factors were determined as previously described. The control versus TTX comparison (Extended Data Fig. 2-3*J*) yielded a significantly different and right-shifted distribution of scaling factors compared with both the control versus control and the TTX versus TTX comparison for all sample sizes (Extended Data Fig. 2-3*A–I*). This validated that even a scaling factor as low as 1.05 would confirm the presence of multiplicative scaling.

The scaling factor is determined by the maximum *p* value obtained from the KS test while comparing the scaled-TTX distribution to the control. The *p* value obtained thus depends on the size of the sample and the variation between the sampled distributions. We have observed a large variation in the *p* values obtained from sampling the same dataset repeatedly (Extended Data Fig. 2-4*A*). We sampled the control and TTX dataset through a range of sample sizes such as 200, 400, 600, 800, 1000, 1200, 1400, 1600, and 1800 as well as multiple times at each sample size, i.e., 100, 500, and 1000 times. We compared the sampled control and TTX dataset within themselves as well as with each other. As we increased the sample sizes the variance of the scaling factor decreased which was consistent with Extended Data Figure 2-2. In order to verify whether the scaling factor that we have calculated is indeed representative, we calculated the “accuracy of scaling factor” for each sampling size and iterations. We plotted the distribution of the scaling factors estimated from repeated sampling for a given sample size and calculated the center of the distribution using Gaussian approximation for each sampling size (Extended Data Fig. 2-4*B*). Second, we calculated the error associated with calculating the center of this distribution by examining the distribution of the error from mean (i.e., the difference between each scaling factor and the mean) and determined the SD of this error (Extended Data Fig. 2-4*C*). The accuracy of scaling factor can thus be represented as the Centre of the Gaussian approximation with the error associated with each sampling. As the sampling size increased, the full-width half maximum of the Gaussian distribution decreased thus decreasing the error associated with the calculation of scaling factors. A sample of the data obtained for a sample size of 1000 data points is displayed as a table (Extended Data Fig. 2-4*D*).

In Kim et al. (2012), mEPSCs were recorded from individual cells and random samples of 100 mEPSCs from each cell was taken, representative of active synapses in that cell. In our experiments, we have collected data from all synapses in a cell contributing to a large heterogeneity in our data. We randomly sampled the total data to do an unbiased analysis, taking into account the heterogeneity. We also sampled equal number of synapses per cell, i.e., 10, 20, 30, and 40 synapses, and compared the control and TTX dataset. At lower sample sizes, the control and TTX dataset showed no significant difference, but as we increased the sample size, the differences between the control and TTX dataset became more apparent and was similar to the random synapses sampled from the total data across the cells.

### Differential scaling of global versus surface pools of GluA2 subunits of AMPA receptors at excitatory synapses

In Figure 1, we looked at multiplicative scaling of synaptic GluA1 and GluA2 subunits of the AMPA receptor with the help of C-terminal antibodies targeted against these subunits, which provided us with an understanding of homeostatic scaling occurring in the global population of GluA1 and GluA2. However, only the surface expressed AMPA receptors would be involved in sensing the released neurotransmitters, and those would be the population of functional AMPA receptors affected by homeostatic scaling. Therefore, we compared multiplicative scaling of the GluA2 subunit using antibodies targeting the N terminus (which identifies only the surface population) and the C terminus (identifying the global population). Primary rat hippocampal neuronal cultures of DIV14 were treated with 2 μM TTX for 48 h followed by immunocytochemistry using N-terminal or C-terminal antibodies against the GluA2 subunit along with Shank2 marking the PSD (Extended Data Fig. 3-1). The distribution of average fluorescence intensities of GluA2 segmented within the Shank2 puncta (which indicates synaptic GluA2) showed significant scaling between the control and TTX conditions for both the global (Fig. 3*A*; *n* = 10 cells, 1893 and 1781 synapses for control and TTX dataset, respectively, *p* < 10^−6^) and the surface (Fig. 3*E*; *n* = 10 cells, 2259 and 2411 synapses for control and TTX dataset, respectively, *p* < 10^−6^) population as determined by the KS test. A random sample of 1000 data points were chosen from the control and TTX dataset, rank ordered and plotted to provide a multiplicative scaling equation for global (Fig. 3*B*; linear fit: y = 0.95x + 1459, *R*^2^ = 0.98) and surface population (Fig. 3*F*; linear fit: y = 1.04x + 0.5, *R*^2^ = 0.99). The scaling factor was determined by scaling down the TTX dataset by multiple arbitrary scaling factors and the scaled-TTX dataset was compared with the control. In order to determine the scaling factor, we repeated the sampling process 100 times and derived an average multiplicative scaling factor of 1.18 ± 0.002 for global GluA2 and 1.08 ± 0.002 for the surface population of GluA2. A representative sampling of the scaling factor and the scaled distribution for global and surface pools of AMPA receptors are presented in Figure 3*C*,*D*, *G*,*H*, respectively. This data displays a correlative scaling for the global and surface population of GluA2 containing AMPA receptors, indicating an increased recruitment of AMPA receptors to the surface in response to homeostatic plasticity. Considering that the C-terminal antibody for GluA2 marks both the intracellular and surface population of GluA2 containing AMPA receptors, it is expected that this scaling factor would be higher in comparison to that obtained exclusively for the surface expressed GluA2. This indicates that in response to homeostatic plasticity there is an increase in total number of receptors at the synapse, but only a fraction of these receptors is targeted to the surface.

10.1523/ENEURO.0407-19.2020.f3-1 Extended Data Figure 3-1 Immunocytochemistry for global and surface GluA2. ***A–D***, Images of GluA2 labelling with the help of C-terminal and (***E–H***) N-terminal antibodies against the GluA2 subunit were obtained on a confocal microscope to indicate the global and surface population of GluA2 containing AMPA receptors. All the images are scaled similarly. Scale bar: 100 μm. Download Figure 3-1, EPS file.

To evaluate the temporal heterogeneity in synaptic scaling we performed longitudinal experiments where the scaling factor in primary neuronal cultures were compared after 24 and 48 h of TTX treatment. Synaptic GluA2 showed multiplicative scaling with a scaling factor of 1.06 ± 0.001 and 1.13 ± 0.001 after 24 and 48 h of TTX treatment, respectively. This indicates that there is an increase in multiplicative scaling of GluA2 containing AMPARs after 48 h of TTX treatment in comparison to 24 h which was in agreement with previous literature (Han and Stevens, 2009). In order to understand the spatial heterogeneity of synaptic scaling we analyzed synapses in the proximal and distal dendritic compartments. Proximal dendrites were always chosen within 66 μm of the cell body and distal dendrites were chosen >132 μm from the cell body. Regions were selected, and image analysis was performed as explained in the methods. We calculated multiplicative scaling factors for them separately and observed that the distal dendrites showed 5.94–7.12% more scaling than the proximal dendrites.

### Scaling down of surface expressed AMPA receptors

Next, we wanted to investigate the scaling down of GluA2 containing AMPA receptors in response to inhibition of inhibitory synapses which leads to an elevation of activity in neuronal cultures. GABA_A_ receptors are one of the major hyperpolarizing ion channels found in inhibitory synapses in hippocampal excitatory pyramidal neurons (Megías et al., 2001). Bicuculline is a well-known competitive antagonist of GABA_A_ receptors which blocks IPSPs, and incubation of neuronal cultures with bicuculline has been shown to cause scaling down of excitatory synapses (Watt et al., 2000; Ibata et al., 2008; Tatavarty et al., 2013). We treated 14 DIV rat hippocampal neurons with 40 μM bicuculline for 48 h. We then fixed and performed immunocytochemistry for Shank2 and surface GluA2. The normalized frequency distribution of GluA2 average intensity segmented within the Shank2 puncta of the bicuculine dataset showed a significant difference from that of the control (Fig. 4*A*; *n* = 10 cells, 1697 and 2988 synapses for control and bicuculline dataset, respectively, *p* < 10^−6^). 1300 synapses were randomly sampled from the control and bicuculline dataset and plotted to provide a multiplicative scaling equation (Fig. 4*B*; y = 0.885x + 67.20, *R*^2^ = 0.99). The average scaling factor calculated by repeating the sampling 100 times was found to be 0.90 ± 0.001. This shows that a multiplicative scaling factor can be reliably calculated for both scaling up and scaling down occurring during homeostatic plasticity.

**Figure 5.**
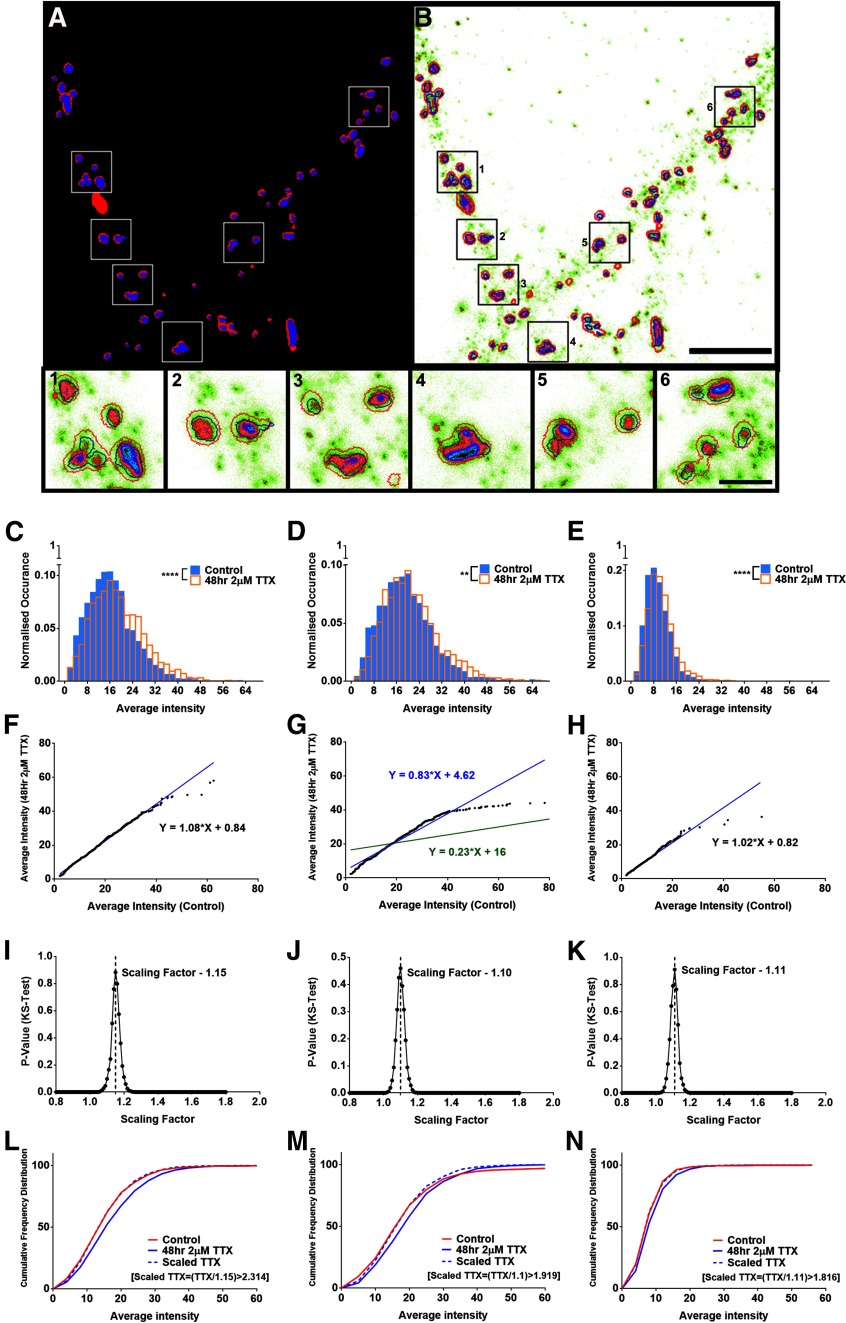
Differential scaling of synaptic AMPA receptor pools within functional zones of an excitatory synapses during homeostatic scaling. ***A***, ***B***, Confocal and STED imaging were performed for Shank2 (***A***) and GluA2 (***B***). ***A***, An adaptive threshold mask was created for Shank2 observed on the synapse (red + blue), perisynapse (red), and PSD (blue) image, and the puncta regions were used to determine different subsynaptic compartments. Analysis scheme for spatial differentiation of functional zones of the synapse using confocal and STED imaging is presented in Extended Data Figure 5-1. ***B***, GluA2 STED image overlaid with red outline indicating the synapse, blue outline indicating the PSD and the region enclosed between the red and blue regions indicating the perisynaptic compartment. ***1–6***, The insets display different zoomed regions of the GluA2 STED image. Scale bars: 4.5 μm (***A***, ***B***) and 1 μm (***1–6***). ***C–E***, Histograms constructed for the average intensity of GluA2 segmented within the Shank2 confocal (***C***), Shank2 STED (***D***), and the area between the Shank2 confocal and STED puncta or the perisynaptic area (***E***) between the control and the TTX dataset showed significant scaling of GluA2 within various subsynaptic compartments (*n* = 2217 and 2401 for GluA2 in the synapse, *p* < 10^−6^, 2694 and 2438 for GluA2 in the PSD, *p* < 10^−4^ and 2276 and 2353 for GluA2 in the perisynapse, *p* < 10^−6^, for control and TTX dataset, respectively, KS test). ***F–H***, A total of 1000 random puncta were chosen from each dataset and the control set was plotted against TTX dataset to provide a scaling equation for the GluA2 scaling within the synapse (***F***; y = 1.08x + 0.84), the PSD [***G***; y = 0.23x + 16.00 (green) and y = 0.83x + 4.62 (blue)], and the perisynapse (***H***; y = 1.02x + 0.82). ***I–K***, Representative scaling factors providing the maximum *p* value between the control and the scaled-TTX dataset was chosen as the multiplicative scaling factor for GluA2 within the synapse, (***I***) in the PSD (***J***) and in the perisynapse (***K***). ***L–N***, Cumulative frequency distribution between the control, TTX, and scaled-TTX dataset showed no significant difference between the control and the scaled-TTX dataset for GluA2 within the synapse (***L***), PSD (***M***), and the perisynapse (***N***). Multiplicative scaling of Shank2 at the PSD is presented in Extended Data Figure 5-2.

10.1523/ENEURO.0407-19.2020.f5-1 Extended Data Figure 5-1 Spatial differentiation of functional zones of the synapse using confocal and STED imaging. ***A***, Shank2 confocal and (***D***) STED images were utilized to identify different subsynaptic compartments in the dendrite. ***B***, Adaptive thresholding method was used to create a mask of the Shank2 confocal and (***E***) the Shank2 STED image. ***C***, Regions indicating the confocal puncta (red) were overlaid on the Shank2 confocal image to determine synaptic regions in the dendrite. ***F***, Regions of the Shank2 STED puncta (blue) were overlaid on the Shank2 STED image to determine the PSD in the dendrite. ***G***, The Shank2 STED mask was subtracted from the Shank2 confocal mask to provide a mask of the perisynaptic region. ***H***, Overlay of the synapse (red) and the PSD (blue) regions. ***1–6***, Different zoomed in regions of the mask and red and blue region to clearly define the distinction in the subsynaptic compartments. Scale bar: 4.5 μm (***A–H***) and 1 μm (***1–6***). Download Figure 5-1, EPS file.

10.1523/ENEURO.0407-19.2020.f5-2 Extended Data Figure 5-2 Multiplicative scaling of Shank2 at the PSD. ***A***, ***B***, Comparison of the distribution of the area (***A***) of Shank 2 puncta (PSD area) and average intensity (***B***) of Shank2 per puncta using STED images from the control and TTX conditions showed scaling after a 48-h treatment (*n* = 2732 and 2442 synapses, for control and TTX dataset, respectively, *p* < 10^−5^ for area and *p* < 10 ^−6^ for average intensity, KS test). ***C***, A total of 1000 random average intensity data points were chosen, rank ordered, and plotted to provide a scaling equation (Y = 0.94*x + 22.31) for the density of Shank2 in the PSD. ***D***, The scaling factor providing the maximum *p* value between control and scaled-TTX datasets was chosen as the multiplicative scaling factor (1.14, *p* = 0.067, KS test) for this repeat. Download Figure 5-2, EPS file.

### Differential scaling of AMPA receptor pools within functional zones of an excitatory synapse

Next, we explored whether the surface expressed AMPA receptors were scaled differentially within functional zones of the synapse. Since the AMPA receptors present in the PSD could directly modulate the signal reception at the synapse, we verified whether the scaling observed at the total synapse was identical to that observed at the PSD. Although previous reports indicate an increase in synaptic AMPA receptors during scaling, it is still unclear if the origin of this increase is due to an increase of receptors in the PSD or in the adjoining perisynaptic compartment, which might serve as a reserve pool to deliver naive receptors. We wanted to differentiate these two scenarios by studying the scaling behavior of the receptors within the respective compartments. We used STED microscopy to examine the subsynaptic organization of surface expressed GluA2 containing AMPA receptors during homeostatic scaling. Shank2 was used as a marker for the PSD, and we estimated the multiplicative scaling factors for GluA2 localized to the synapse, the PSD, and the perisynapse.

Primary rat hippocampal neurons of DIV14 were treated with 2 μm TTX for 48 h. Immunocytochemistry was performed for surface GluA2 and Shank2, and dendritic regions were imaged using confocal and STED microscopy (Fig. 5; Extended Data Fig. 5-1). The Shank2 confocal image was sampled the same way as the STED image. Because of the inability of confocal microscopy to differentiate the different subsynaptic zones, confocal image of Shank2 was used to determine the regions indicating the excitatory synapse on the dendrite. The Shank2 STED image was used to determine the PSD. The region on the Shank2 confocal image excluding the STED puncta were used to determine the perisynaptic region (Fig. 5*A*; Extended Data Fig. 5-1). Shank2 STED images were analyzed for changes in the characteristics of PSD in control and TTX dataset. The PSD area and average intensity of Shank2 in the PSD showed significant scaling after TTX treatment (Extended Data Fig. 5-2*A*,*B*). The scaling of Shank2 at the PSD was observed to be multiplicative with an average scaling factor of 1.18 ± 0.002 (Extended Data Fig. 5-2*C*,*D*). We have also observed a significant increase in the Shank2 confocal area (which indicates the synapse area) and the total intensity of GluA2 segmented within the Shank2 confocal and STED masks (data not shown). The average intensity of GluA2 segmented within the Shank2 confocal and STED images were used to determine GluA2 scaling within various subsynaptic compartments (Fig. 5*B*,*1–6*). A histogram plotted for the frequency distribution of average intensity of GluA2 in the synapse, PSD and perisynaptic region between control and TTX conditions indicated significant scaling with *p* values of *p* < 10^−6^, *p* < 10^−4^, and *p* < 10^−6^, respectively (*n* = 2217 and 2401 for GluA2 in the synapse, 2694 and 2438 for GluA2 in the PSD and 2276 and 2353 for GluA2 in the perisynapse, for control and TTX dataset, respectively; Fig. 5*C–E*). 1000 puncta were chosen randomly from the control and TTX dataset, rank ordered and plotted as control versus TTX to provide a scaling equation for GluA2 at the synapse (linear fit: y = 1.08x + 0.84, *R*^2^ = 0.99), within the PSD (linear fit: y = 0.23x + 16.00, *R*^2^ = 0.51 (Fig. 5*G*, green), and at the perisynapse (linear fit: y = 1.02x + 0.82, *R*^2^ = 0.96; Fig. 5*F–H*). A small number of datapoints showed a large variance from majority of the data in the control dataset for GluA2 within PSD, leading to an aberrant nature for the slope in the rank-ordered plot (Fig. 5*G*, green). Therefore, the *x*-axis was curtailed at an average intensity of 80 to obtain a new linear fit (linear fit: y = 0.83x + 4.62, *R*^2^ = 0.94; Fig. 5*G*, blue). The sampling was repeated 100 times to determine an accurate scaling factor for the average intensity of GluA2 at the synapse, PSD, and perisynapse as 1.13 ± 0.002, 1.09 ± 0.002, and 1.13 ± 0.002, respectively. The representative scaling factors for the synapse, PSD and perisynapse are shown in Figure 5*I–K*. The TTX dataset was scaled down with the calculated scaling factor and the cumulative frequency distribution was plotted between the control, TTX and scaled-TTX dataset, indicating no significant difference between the control and the scaled-TTX dataset for GluA2 within the synapse, PSD or at the perisynapse (Fig. 5*L–N*). The correlative scaling factor between GluA2 at the synapse and perisynapse indicated an increased recruitment of GluA2 to the perisynapse in comparison to the PSD. This indicated that the receptors at the perisynaptic compartment could act as a reserve pool of naive receptors, which would then be trafficked on an activity dependent manner to the PSD.

## Discussion

Homeostatic scaling is characterized by direct alteration of the density of functional AMPA receptors at excitatory synapses. However, little is known about the changes that govern synaptic molecules which associate with AMPA receptors such as scaffolding molecules, cell adhesion molecules, transmembrane AMPA receptor regulatory proteins and other signaling molecules. Indeed, these molecules control the localization, trafficking and post-translational modification of receptors, thus directly influencing the receptor function during homeostatic plasticity and local plasticity mechanisms including long-term potentiation/depression (Vitureira and Goda, 2013; Fernandes and Carvalho, 2016; Henley and Wilkinson, 2016; Diering and Huganir, 2018). Here we demonstrate a simple immunocytochemical method based on rank ordered analysis to quantitatively characterize multiplicative homeostatic scaling induced by activity blockade in neuronal cultures. We use this analysis paradigm to extract scaling factors for various AMPA receptor subunits and some of the key presynaptic and postsynaptic proteins. We observed that there is significant scaling on the content of both presynaptic and postsynaptic scaffolding molecules on neuronal activity blockade. This is consistent with the observation that induction of homeostatic scaling in older cultures results in the augmentation of both the frequency and amplitude of the postsynaptic response when measured using electrophysiological techniques (Han and Stevens, 2009). Increase in the content of presynaptic scaffolding molecules such as Bassoon indicates strengthening of the cytomatrix of the active zone in the presynapse. Higher levels of cytomatrix proteins such as Bassoon and Piccolo have been correlated with faster reloading of presynaptic vesicles modulating both the frequency and amplitude of EPSCs at central synapses (Hallermann et al., 2010; Mukherjee et al., 2010; Kittel and Heckmann, 2016). An alteration in the synaptic content of postsynaptic scaffolding molecules such as PSD95 and Shank is also known to affect the basal synaptic transmission affecting the expression, localization, and retention of AMPA receptors at excitatory synapses (Henley and Wilkinson, 2016; Diering and Huganir, 2018). We observed augmentation of scaling factor for AMPA receptor subunits on activity blockade, confirming an increase in the synaptic numbers for both GluA1 and GluA2 containing AMPA receptors. However, activity-dependent subunit, GluA1 showed a higher scaling in comparison to GluA2. Although there is an increase in both GluA1 and GluA2 subunits, there might be a preferential recruitment of AMPA receptors containing both GluA2/GluA1 subunits in comparison to AMPA receptors containing GluA2 with other subunits. While comparing the control and scaled-TTX distributions, the GluA1 data alone showed a different statistical result while using the AD test compared with the KS test. This shows that GluA1 may undergo homeostatic scaling that is not purely multiplicative. This could be because of the differences in the subunit stoichiometry of AMPA receptors. (Diering and Huganir, 2018). Furthermore, it was observed that synapses localized to distal dendrites exhibit augmented scaling compared with their counterparts in proximal dendrites (Smith et al., 2003). Such a scaling up of synapses in distal dendrites might be a requirement to compensate for cable filtering, where the synapses would need to ensure the availability of more receptors for the EPSPs to reach the soma. Since the archetypal connectivity is not maintained in primary neuronal cultures, it remains to be seen whether this is the case is intact brain slices where the morphology and connectivity is preserved.

The results obtained from rank order analysis of microscopy images is consistent with the electrophysiological data which has used comparable statistics to evaluate mEPSC recordings from control and TTX-treated primary neuronal cultures (Kim et al., 2012). However, the scaling factor obtained for AMPA receptor subunits by immunocytochemistry was lower than that obtained for amplitude scaling of synaptic responses by electrophysiological data. These differences could be attributed to the differing methods for data collection in these techniques. Electrophysiological recording involves recording of mEPSC traces for a finite amount of time. Same synaptic responses can be recorded multiple times shifting the bias toward more active synapses during the time of recording. Contrary to electrophysiological recordings, observing random image fields by immunocytochemical labeling would result in unbiased inclusion of all the synapses in the field of view resulting in larger heterogeneity of synapses available for evaluation. The method that we have implemented could also be combined with electrophysiological recordings, thus bridging the gap between these two techniques. Another major strength of this method is the ability to show using random sampling of synapses that the variance in the calculation of the scaling factor reduces asymptotically as the number of synapses used for the analysis increases. Sampling for rank order analysis could be performed with half the number of detected synapses to yield an accurate scaling factor. Furthermore, to confirm that the small scaling factors we obtained were indeed indicative of multiplicative scaling, we asked the question of how small a scaling factor could be considered significant. To address this, we randomly subdivided a control dataset of fluorescence intensities into two datasets and calculated a scaling factor between these control subsets. A similar procedure was adopted for the TTX dataset as well. We were able to show that a scaling factor as low as 1.05 between the control and TTX dataset was significantly greater than what was obtained from within group comparison of the control or TTX datasets, proving that this method is indeed a robust estimator for multiplicative homeostatic scaling.

The neuronal synapse is a very dynamic and organized structure. The instantaneous receptor population can be divided into pools of receptors localized within specific functional zones of the synapse. We observed that these different pools of AMPA receptors are differentially scaled. For example, the global pool of AMPA receptors was scaled higher than the surface AMPA receptors. This differential scaling could arise since TTX suppresses action potentials which would significantly affect the large pool of receptors waiting to be inserted into the synaptic membrane during synaptic activity. Previous studies have indicated that AMPA receptors can be immobilized both in the postsynaptic and perisynaptic compartments and the equilibrium between these subsynaptic zones can be maintained by lateral diffusion (Chen et al., 2018; Scheefhals and MacGillavry, 2018; Humeau and Choquet, 2019). With the help of super-resolution imaging, we were able to discern different pools of AMPA receptors associated with different functional zones of the synapse. Advanced microscopy paradigms with improved 2D and 3D resolution might give a better perspective of molecular distribution of receptors in the PSD; enabling a better discrimination of synaptic versus extrasynaptic pools on the functional zones of the synapse. Although the rank order analysis does not consider nanoscale distributions of the molecules inside the functional zones, it remains a very good tool to evaluate the characteristics of AMPA receptor pools in the functional zones of the synapse. Using the rank order analysis, scaling in the perisynaptic compartment was found to be higher than that of the PSD compartment. Although there was an increase in size of the PSD, and density of scaffolding molecules in the PSD after induction of homeostatic scaling, a larger number of these synapses reached saturation for AMPA receptor density in comparison with the control. This indicates that there is a limiting size for the PSD and the maximum number of receptors that can be accommodated per PSD. We observed that the AMPA receptor population at the PSD for the TTX distribution saturated at higher intensities, indicating that there was increased packing of receptors into the PSD. Furthermore, the PSD has a comparatively smaller area than the spine which accounts for the saturation of receptor molecules in the PSD compared with that in the spine. Irrespective of this size constraint for the PSD, the perisynaptic compartment showed higher scaling. A larger number of receptors were localized to a reserve pool in the perisynaptic compartment where active processes such as endo- and exocytosis occur. This increase in reserve pool of receptors could access the PSD immediately by lateral diffusion on activity and modulate the postsynaptic response. These results confirm a higher recruitment of GluA2 to the perisynapse on homeostatic scaling. This could also be due to the constraints imposed on the size and molecular content of the PSD. Our observations are on par with previous studies on LTP, showing that AMPA receptor insertion first occurs at the perisynapse from whence the receptors laterally diffuse to the PSD (Vitureira and Goda, 2013; Bailey et al., 2015; Fernandes and Carvalho, 2016; Henley and Wilkinson, 2016; Diering and Huganir, 2018), suggesting that mechanisms may be conserved between homeostatic and long-term plasticity.

## Conclusion

It is currently known that there can be dendritically localized forms of homeostatic scaling (Yu and Goda, 2009; Fernandes and Carvalho, 2016; Barnes et al., 2017). The local plasticity mechanisms also involve an increase or decrease in the strength of individual synapses by recruiting more receptors and receptor associated proteins to the synapses. The rank order analysis to calculate the relative scaling factor from immunolabelled images can be used to determine the dendritic or synaptic contributions to different forms of plasticity. Since immunolabelling *in vitro* or *in vivo* is a routine technique that is performed across many laboratories, this analysis protocol can be used as a routine tool to measure synaptic increase of proteins of interest and thus bring in novel information regarding protein machinery involved in different forms of synaptic plasticity. Furthermore, the use of rank order analysis of microscopic images together with electrophysiology would enable us to address the dynamic nature of synaptic plasticity and provide insights into the molecular basis of plasticity at the finest detail possible.

## Synthesis

Reviewing Editor: Niraj Desai, NINDS Decisions are customarily a result of the Reviewing Editor and the peer reviewers coming together and discussing their recommendations until a consensus is reached. When revisions are invited, a fact-based synthesis statement explaining their decision and outlining what is needed to prepare a revision will be listed below. The following reviewer(s) agreed to reveal their identity: Jary Delgado.

The reviewers and editor were generally enthusiastic about this study, which offers a useful means of determining scaling factors after homeostatic plasticity, not only for synaptic receptors but for other proteins associated with synaptic transmission. However, they believe the manuscript could be strengthened in several ways, principally having to do with characterizing the accuracy and range of applicability of the authors’ methods.

1) STATISTICAL TESTS

How accurate is the scaling factor determined by these methods? Although the rank-ordered fluorescence intensity approach adopted here seems to work in general, some of the provided data need more clarification. In Fig1N, the scaled TTX curve is claimed by the authors to be indistinguishable from control but the distributions look quite different. It would be worthwhile to test the data with alternative statistical tests that are more sensitive to differences in the tails of the distribution (e.g., Anderson Darling). Since the result of Fig1N was also the largest scaling effect, this observation suggests that the larger the response the greater the deviation between the scaled trace and the control trace (i.e., the larger the error in determining the scaling factor). The use of an extremely stringent threshold for significance may be causing a false negative.

A related point: what is the relationship between the max p value and the accuracy of the scaling factor? In some of the analyzes, one sees that the max p values differ by orders of magnitude. In general, it seems that smaller effect sizes correlate with larger max p values. It would be very useful - and an important advance - if the authors could devise a means of estimating scaling factor with a confidence score attached to the p value.

2) CELL BY CELL

The authors report the number of cells used in each analysis and the number of synapses but not the number of synapses per cell or the variability from cell to cell. Physiological studies of multiplicative scaling using mEPSCs (including Kim et al., 2012) attempt to counter the possibility of a cell bias by drawing an equal number of events from each cell. Why not do that here?

It is also important to report the mean values per cell and to do some of the statistics on these numbers rather than on the global distributions - especially statistics contrasting control and treatment groups. Presently the authors use the Kolmogorov-Smirnov test to report, for example, that the control and TTX groups of Fig1K differ with a p value of less than 10E-5. If one simply looks at that figure, this p value is not believable. Maybe the two groups are different, but the uncertainty in that conclusion is much greater than p=10E-5 would imply. Likewise, the manuscript presently claims that the control and TTX groups of Fig2A differ with a p value of just over 10E-32. While the two distributions of Fig2A look much more distinct than those of Fig1K, this p value borders on absurd. The Kolmogorov-Smirnov test is notorious for producing outlandish results when the N value gets large; the authors should be mindful of that problem and do a “by cell” analysis instead. Moreover, they should compare the size of the effect of TTX treatment derived from a mean value per cell analysis to that derived from the multiplicative scaling method, as they should be about the same order of magnitude.

A related point: some scientists who study synaptic scaling have reported much smaller effects after 24 h of activity manipulation than after 48 h. For some of the experiments, the authors use 48 h but for others they use 24 h. Why? This is never explained, and the two durations are never contrasted.

3) SYNAPTIC VS EXTRASYNAPTIC

The scaling factors determined by the rank-ordered analysis seem much smaller than those obtained by conventional methods. mEPSC data are dominated by synaptic glutamate receptor responses and may not have much contribution from extra synaptic receptors that are not directly opposed to release sites. This could be another important factor that could be adding noise to the determination of a scaling factor based on immunostaining (which is integrating synaptic and extra synaptic responses).

4) SCALING DOWN

It is important to determine if this technique can be used to estimate the scaling factor for scaling down as well. This is because the effect size for scaling down is much smaller compared to scaling up. It would therefore be interesting to know if this technique is able to pick up differences between control and scaling down conditions in order to determine its range of applicability.

5) CHANGES IN AREA

It would be useful to see an analysis of the changes in area size of the puncta, before and after TTX treatment for the dataset (like what was done for the STED data). Since the data of Fig1L, 2C, and 4 G seem to suggest a saturation of glutamate receptor levels in the TTX conditions (as the authors point out in the discussion), it is possible that intensity signal is spread over a larger area with the same saturation in density. Therefore, looking at the total fluorescence intensity as opposed to average, could be of value.

6) RAT VS MOUSE

The authors use different thresholding criteria for the rat data and the mouse data, but they don’t explain why. They have to explain.

7) COMPARISON WITH LITERATURE

While the GluA1 and GluA2 numbers reported here are on par with the literature, the PSD95 number is rather different than the 1.44 increase in puncta intensity reported by Sun and Turrigiano (2011). Why is that? The authors must at least address the discrepancy in discussion.

8) DISTANCE FROM SOMA

The authors never mention how far analyzed synapses were from the soma - but they should. It is important to know how scaling depends on dendritic distance - and whether that affects scaling. Given the kind of images the authors must possess, they should address this issue and include relevant images with this analysis.

9) SHANK2

The authors use the postsynaptic density protein Shank2 in their study, but never explain why.

10) AVERAGE INTENSITY

On the histograms, what is presently called “average intensity” might better be called “cluster intensity” with some indication that these are arbitrary units.

## Author Response

Synthesis Statement for Author (Required):

The reviewers and editor were generally enthusiastic about this study, which offers a useful means of determining scaling factors after homeostatic plasticity, not only for synaptic receptors but for other proteins associated with synaptic transmission. However, they believe the manuscript could be strengthened in several ways, principally having to do with characterizing the accuracy and range of applicability of the authors’ methods.

We are very grateful to the editor and to the reviewers for evaluating the manuscript positively and for their insightful comments to strengthen the manuscript. We have performed all the additional analyses, experiments, and quantifications to answer the queries raised by reviewers. We have also added appropriate additional figures to the main manuscript and supplementary resources to address the queries raised in the synthesis statement to authors. We believe that the comments have improved the manuscript in its scientific content and technical execution. We thank the editor and reviewers again, and hope our responses are satisfactory and meet their expectations.

1) STATISTICAL TESTS

How accurate is the scaling factor determined by these methods? Although the rank-ordered fluorescence intensity approach adopted here seems to work in general, some of the provided data need more clarification. In Fig1N, the scaled TTX curve is claimed by the authors to be indistinguishable from control but the distributions look quite different. It would be worthwhile to test the data with alternative statistical tests that are more sensitive to differences in the tails of the distribution (e.g., Anderson Darling). Since the result of Fig1N was also the largest scaling effect, this observation suggests that the larger the response the greater the deviation between the scaled trace and the control trace (i.e., the larger the error in determining the scaling factor). The use of an extremely stringent threshold for significance may be causing a false negative.

The reviewers have raised a very valid point. We had used KS test, which showed the scaled TTX to be not significantly different from the control data. Reviewers were right to point out the use of an alternative statistical analysis such as the Anderson-Darling (AD) test to study the differences between these distributions. In statistics, the AD test is mostly used to determine the normality of a distribution in commonly used data analysis software. However, in order to compare two distributions by AD test we followed the protocols explained in ‘Comparing distributions: the two-sample Anderson-Darling test as an alternative to the Kolmogorov-Smirnoff test’ by Sonja Engmann and Denis Cousineau and the ‘K-Sample Anderson-Darling Tests of Fit, for Continuous and Discrete Cases’ by F.W. Scholz and M.A. Stephens (Scholz and Stephens, 1987; Engmann and Cousineau, 2011). We designed our own code in Python to perform a 2 sample AD test on several of our datasets. Unlike the KS test, AD gives us a set of critical values (ie. 0.325, 1.226, 1.961, 2.718, 3.752) for different confidence intervals (ie. 75, 90, 95, 97.5 and 99% respectively). The output is a statistic value that must be compared against the critical values in order to determine its significance. The statistic value must be higher than the critical value for the data to be significant.

We used the AD test to compare the TTX and scaled TTX distributions. We observed that except for the GluA1 data, the AD test provided a statistic value far below the critical values for most of our data including GluA2, confirming that the scaled TTX distributions were not significantly different from the control distributions. For GluA1, the statistic value for control versus scaled TTX was found to be greater than the critical value, but less than that of the control versus TTX dataset. We used the AD test to calculate the multiplicative scaling factor by determining the scaling factor at which the test statistic for control vs scaled TTX showed the smallest value for GluA1, and we obtained a scaling factor similar to the one obtained with the help of the KS test indicating that the scaling factor is indeed comparable across the statistical tests. Based on the results of AD test alone, the GluA1 might undergo a scaling that is not purely multiplicative. This could be because of the differences in the subunit stoichiometry of AMPA receptors.

The statistical method relevant to the AD test have been added to the ‘Materials and Methods’ section of the manuscript under the ‘Statistics’ sub-section. The results indicated by the AD test for GluA1 and GluA2 from figure 1 have been added as the fourth paragraph under the ‘Multiplicative scaling of synaptic proteins determined using Rank order analysis’ sub-section of the ‘Results’ section in the manuscript.

A related point: what is the relationship between the max p value and the accuracy of the scaling factor? In some of the analyzes, one sees that the max p values differ by orders of magnitude. In general, it seems that smaller effect sizes correlate with larger max p values. It would be very useful - and an important advance - if the authors could devise a means of estimating scaling factor with a confidence score attached to the p value.

The scaling factor is determined by the maximum p-value obtained from KS test, while comparing the scaled TTX distribution to the control. The analysis that we perform does not rely on the absolute p values, but where the p-value attains the maximum possible value as we compare the distributions. The p-value obtained thus depends on the size of the sample and the variation between the sampled distributions. We have observed a large variation in the p-values obtained from sampling the same dataset repeatedly (Response to reviewers Figure 1A). Despite such a variability in the p-values, it was important to observe the variability of scaling factors associated with each data set. Here, we sampled the control and TTX dataset through a range of sample sizes such as 200, 400, 600, 800, 1000, 1200, 1400, 1600 and 1800 as well as multiple times at each sample size ie. 100, 500 and 1000 times. We compared the sampled control and TTX treated data within themselves as well as between each other. As we increased the sample sizes the variance of the scaling factor decreased, which was consistent with extended data figure 2 which we represent for 100 iterations. In order to verify if the scaling factor that we have calculated in the paper was representative, we calculated the “accuracy of scaling factor” for each sampling size and iteration. For this, we first plotted the occurrence of the scaling factor and calculated the centre of the distribution using Gaussian approximation for each sampling size (Response to reviewers Figure 1B). Secondly, we calculated the error associated with calculating the centre of this distribution by studying the distribution of the error from mean for each scaling factor and determined the standard deviation of this error (Response to reviewers Figure 1C). The “accuracy of scaling factor” can thus be represented as the Centre of the Gaussian approximation with the error associated with each sampling. As the sampling size increased, the full-width half maximum of the Gaussian distribution decreased, thus decreasing the error associated with the calculation of the scaling factors. A sample of the data obtained for a sample size of 1000 data points is displayed as a table (Response to reviewers Figure 1D).

Thus we can confirm that the scaling factor that we have independently calculated for each data always falls in the “accuracy of scaling”. This further confirms that the scaling factor that is generated is independent of the absolute p-value, but is dependent on the maximum p-value (a relative quantity) of every distribution.

Response to reviewers Figure 1 has been included in the manuscript as extended data figure 4. The results corresponding to the ‘accuracy of scaling’ detailed here has been included as the second paragraph under the ‘Effect of sampling on the accuracy of measurement of the scaling factor’ sub-section of the ‘Results’ section in the manuscript.

Response to reviewers Figure 1: Accuracy of Scaling in homeostatic plasticity of Shank2 at 48 hours.

(A) The maximum p-values obtained while comparing 1000 randomly sampled synapses from the control and treated distribution during the calculation of scaling factor repeated 500 times. (B) Gaussian fits of the normalized frequency distribution of scaling factors obtained while comparing the control and TTX datasets for each sampling size repeated 500 times. (C) Gaussian fit of the normalized frequency distribution of the error of the scaling factors obtained in figure 1B. (D) Tabular column detailing the average scaling factor and standard deviation of error obtained for 1000 randomly sampled synapses from the control and TTX dataset repeated 100, 500 and 1000 times.

2) CELL BY CELL

The authors report the number of cells used in each analysis and the number of synapses but not the number of synapses per cell or the variability from cell to cell. Physiological studies of multiplicative scaling using mEPSCs (including Kim et al., 2012) attempt to counter the possibility of a cell bias by drawing an equal number of events from each cell. Why not do that here?

It is also important to report the mean values per cell and to do some of the statistics on these numbers rather than on the global distributions - especially statistics contrasting control and treatment groups. Presently the authors use the Kolmogorov-Smirnov test to report, for example, that the control and TTX groups of Fig1K differ with a p value of less than 10E-5. If one simply looks at that figure, this p value is not believable. Maybe the two groups are different, but the uncertainty in that conclusion is much greater than p=10E-5 would imply. Likewise, the manuscript presently claims that the control and TTX groups of Fig2A differ with a p value of just over 10E-32. While the two distributions of Fig2A look much more distinct than those of Fig1K, this p value borders on absurd. The Kolmogorov-Smirnov test is notorious for producing outlandish results when the N value gets large; the authors should be mindful of that problem and do a “by cell” analysis instead. Moreover, they should compare the size of the effect of TTX treatment derived from a mean value per cell analysis to that derived from the multiplicative scaling method, as they should be about the same order of magnitude.

The reviewers have mentioned an interesting point. In Kim et al. 2012, mEPSCs were recorded from individual cells and random samples of 100 mEPSCs from each cell were taken, representative of active synapses in that cell (Kim et al., 2012). In our experiments, we have collected data from all synapses in a cell contributing to a large heterogeneity in our data. We randomly sampled the total data to do an unbiased analysis, taking account of the heterogeneity. To address the reviewers’ comment of sampling equal number of synapses per cell, we sampled 10, 20, 30 and 40 synapses, and compared the control and TTX data as shown in Response to reviewers Tables 1 and 2. At lower sample sizes, the control and TTX data showed no significant difference, but as we increased the sample size, the differences between the control and TTX data became more apparent. Response to reviewers Tables 1 and 2 show the p values obtained from KS test, comparing Control vs Control, TTX vs TTX and Control vs TTX for different sample sizes.

Response to reviewers Table 1 and 2: Sampling of synaptic GluA2 - Confocal and STED data.

Varying sizes of random samples of synapses (ie. 10, 20, 30 and 40 synapses) were taken from each image and cumulated. The different data were compared among themselves as well as between each other at each sampling size with the help of the 2 sample KS test. The significance level was set at p = 0.001. The relationship between number of stars and p-value can be found in the materials and methods.

We agree with the reviewers that taking a constant number of synapses across cells may be a better reflection of the previously used methods to study multiplicative scaling (Ibata et al., 2008; Gainey et al., 2009; Sun and Turrigiano, 2011). We also compared the multiplicative scaling factor derived from a mean value per cell analysis to that derived from the sampling of random synapses method. We examined the cell-wise averages of average intensity of GluA1 puncta shown in figure 1A and found that the mean GluA1 average intensity of TTX-treated cells showed significant scaling in comparison to that of the control cells, corresponding to a scaling of 1.12 {plus minus} 0.028 (control - 1.00 {plus minus} 0.027, t(19) = 2.94, p=0.008, unpaired t-test). We observed that this scaling factor was lesser than the one obtained from the random sampling method ie. 1.30 {plus minus} 0.002. The cell-wise average value provides equal weightage for every synapse in a cell without taking synaptic heterogeneity into account. In comparison, repeatedly sampling 80% of all the synapses and calculating a scaling factor suitable for each repeat takes into account the inherent variation observed across single synapses.

Synapses are heterogeneous structures with varying number of similar molecules between them. Thus they follow a non-normal distribution in their morphological/molecular characteristics. Additionally, averaging all synapses per cell is limiting because it averages effect sizes across all synapses within a cell and the diversity in initial synapse strength is diluted. The scaling factor calculated from this per cell average may not cover the synaptic heterogeneity in the synapses, as compared to the scaling factor calculated from all synapses treated individually. The per-cell average maybe useful to detect the presence of scaling as an overall effect, but it is not reflective of true multiplicative changes within synapses. These additional analyses and explanations have been incorporated to the results section in the manuscript.

The results of the cell-wise average analysis has been added as the fifth paragraph under the ‘Multiplicative scaling of synaptic proteins determined using Rank order analysis’ sub-section of the ‘Results’ section in the manuscript. The unpaired t-test used to compare the cell-wise averages between the control and TTX dataset has been explained in the ‘Statistics’ sub-section of the ‘Materials and Methods’ section in the manuscript. The results from the equal sampling of synapses has been included as the third paragraph under the ‘Effect of sampling on the accuracy of measurement of the scaling factor’ sub-section of the ‘Results’ section in the manuscript.

A related point: some scientists who study synaptic scaling have reported much smaller effects after 24 h of activity manipulation than after 48 h. For some of the experiments, the authors use 48 h but for others they use 24 h. Why? This is never explained, and the two durations are never contrasted.

We are extremely grateful to the reviewers for raising this concern. Han et al. 2009 indicated that there is homeostatic scaling of mEPSC frequency after 24 hours of TTX treatment, and a scaling of both mEPSC frequency and amplitude following 48 hours of TTX treatment in DIV 14 primary neurons (Han and Stevens, 2009). Using our approach, we longitudinally examined scaling in the same cultures following TTX treatment for 24 and 48 hours. Synaptic GluA2 showed multiplicative scaling with a scaling factor of 1.06{plus minus}0.001 and 1.13{plus minus}0.001 for 24 and 48 hours of TTX treatment, respectively. This indicates that there is an increase in multiplicative scaling of postsynaptic markers after 48 hours of TTX treatment in comparison to 24 hours, conforming with the results shown in Han et al. 2009.

The results obtained for the longitudinal examination of synaptic GluA2 after 24 and 48 hours of TTX treatment has been included in the second paragraph under the ‘Differential scaling of global vs surface pools of GluA2 subunits of AMPA receptors at excitatory synapses’ sub-section of the ‘Results’ section of the manuscript.

Response to reviewers Figure 2: ‘Longitudinal verification of multiplicative scaling in synaptic GluA2’

(A&B) Cumulative frequency distribution of sampled average intensity of the control, TTX and scaled TTX (TTX/scaling factor>minimum of control) distributions of synaptic GluA2 for (A) 24 hours TTX treatment and (B) 48 hours TTX treatment. There is no significant difference between control and scaled TTX population for both the treatment conditions.

3) SYNAPTIC VS EXTRASYNAPTIC

The scaling factors determined by the rank-ordered analysis seem much smaller than those obtained by conventional methods. mEPSC data are dominated by synaptic glutamate receptor responses and may not have much contribution from extra synaptic receptors that are not directly opposed to release sites. This could be another important factor that could be adding noise to the determination of a scaling factor based on immunostaining (which is integrating synaptic and extra synaptic responses).

We agree with the reviewers that the scaling factors determined by immunostaining are different from those obtained from conventional mEPSC analysis, due to the nature of the synapses (silent or active) and localization of receptors (extrasynaptic or synaptic receptors) picked up by either method. We have added this point to our discussion of differences between electrophysiological and immunocytochemical estimation of scaling factors.

We have used 2D STED to differentiate between synaptic and perisynaptic pools of AMPA receptors in figure 4. The use of 3D STED/other 3D super resolution paradigms (with better resolution in Z axis) would be able to better differentiate between synaptic and extra synaptic pools of GluA2 containing AMPA receptors based on 3D volume of a synapse. We agree that addressing the spatial segregation of synaptic molecules with better resolution might improve the accuracy of this analysis. The use of advanced microscopy techniques to better understand the molecular distribution of the synapse has been discussed in the third paragraph of the ‘Discussion’ section in the manuscript.

4) SCALING DOWN

It is important to determine if this technique can be used to estimate the scaling factor for scaling down as well. This is because the effect size for scaling down is much smaller compared to scaling up. It would, therefore, be interesting to know if this technique is able to pick up differences between control and scaling down conditions in order to determine its range of applicability.

We thank the reviewers for this question. Bicuculline is a known competitive antagonist of GABAa receptor, which is often used to study homeostatic scaling down of mEPSCs in primary neuronal cultures (Watt et al., 2000). We treated DIV 14 rat hippocampal neurons with 40μM bicuculline for 48 hours and performed GluA2 and Shank immunolabelling to study scaling down of synaptic GluA2. We observed scaling down of synaptic GluA2 with a scaling factor of 0.90{plus minus}0.001. Our immunocytochemical method of estimation is able to successfully pick up differences between scaling up and scaling down.

The scaling down results has been added as the ‘Scaling down of surface expressed AMPA receptors’ sub-section under the ‘Results’ section of the manuscript with its corresponding figure ie. figure 4. The bicuculline treatment protocol has been included under the ‘Homeostatic Plasticity Induction Protocol’ sub-section of the ‘Materials and Methods’ section in the manuscript.

Response to reviewers Figure 3: ‘Scaling down of synaptic GluA2’

(A) Cumulative frequency distribution of sampled average intensity of synaptic GluA2 in control, TTX and scaled TTX. The scaling factor is 1.08{plus minus}0.002 (B) Cumulative frequency distribution of sampled average intensity of synaptic GluA2 in control, Bicuculline and scaled Bicuculline. The scaling factor is 0.9{plus minus}0.001. There was no significant difference observed between the control and scaled datasets.

5) CHANGES IN AREA

It would be useful to see an analysis of the changes in area size of the puncta, before and after TTX treatment for the dataset (like what was done for the STED data). Since the data of Fig1L, 2C, and 4 G seem to suggest a saturation of glutamate receptor levels in the TTX conditions (as the authors point out in the discussion), it is possible that intensity signal is spread over a larger area with the same saturation in density. Therefore, looking at the total fluorescence intensity as opposed to average could be of value.

As per the suggestion by the reviewers, we have studied the changes in area of the post synaptic density marker by confocal (Response to reviewers figure 4A) and STED (Extended data figure 7A). Both of these data showed a significant increase in the number of larger synapses. We also observed a significant increase in the total intensity of AMPA receptor subunit GluA2 in the confocal (Response to reviewers figure 4B) and STED masks (Response to reviewers figure 4C). This data is in resonance with the saturation of AMPA receptor levels at the synapse after TTX treatment. Furthermore, the post synaptic density (masked by the STED image) has a comparatively smaller area than the spine (masked by the confocal image). This accounts for the saturation of receptor molecules in the PSD as shown by figure 5G, compared to the spine as shown by figure 5F.

The changes observed in the area of PSD and intensity of Shank2 in the spine and PSD have been described in the second paragraph of the ‘Differential scaling of AMPA receptor pools within functional zones of an excitatory synapse’ sub-section of the ‘Results’ section with the help of extended data figure 7 in the manuscript. The implications of these results have been discussed in the third paragraph of the ‘Discussion’ section in the manuscript.

Response to reviewers Figure 4: (A) Frequency distribution of puncta area measured with the help of Shank2 showed a significant difference between the control and TTX dataset. (B&C) Comparison between the distribution of total puncta intensity of GluA2 segmented within the spine (indicated by Shank2 confocal mask) (B) and the PSD (indicated by Shank2 STED mask) (C). Both showed a significant change between the control and TTX dataset.

6) RAT VS MOUSE

The authors use different thresholding criteria for the rat data and the mouse data, but they don’t explain why. They have to explain.

We appreciate the reviewer’s concern in pointing out a parameter that we missed to report in the integrated morphometry analysis. The mouse data was acquired with a sampling of 90nm per pixel. The rat data, on the other hand, was acquired using a confocal mode of 2D STED microscope, where the confocal images were oversampled with a sampling size of 15nm to keep the sampling the same as the STED data. Taking the pixel area into account, a minimum area threshold of 0.02 μm2 was given for the mouse data and 0.038 μm2 was given for the rat data. Additionally, we had used an additional minimum length filter of 0.18μm (to account for resolution limit in confocal microscope) in the Integrated morphometry analysis for both the data. Thus the final cut off values for the minimum areas from both datawould be very close.To verify that this protocol was appropriate, we analysed the rat synaptic GluA2 data by reducing the lower area threshold to 0.02 μm2 which provided a scaling factor of 1.14{plus minus}0.0008, and was similar to the scaling factor obtained with an area threshold of 0.038 μm2 which was 1.15{plus minus}0.0005.

We apologize for this error and have now clarified this in the ‘Image Analysis’ sub-section of the ‘Materials and Methods’ section in the manuscript.

7) COMPARISON WITH LITERATURE

While the GluA1 and GluA2 numbers reported here are on par with the literature, the PSD95 number is rather different than the 1.44 increase in puncta intensity reported by Sun and Turrigiano (2011). Why is that? The authors must at least address the discrepancy in discussion.

Quoting Sun and Turrigiano 2011, “The total fluorescence intensity of puncta was measured using the “granularity” function in MetaMorph” which calculated the total fluorescence intensity of the puncta (Sun and Turrigiano, 2011). We have utilized average intensity for our scaling factor calculations, as it reflects the density of molecules at the synapse. As the reviewers suggested, we have measured the scaling factor using the total puncta intensity of PSD95 in both mouse and rat hippocampal neurons after 48 hours of TTX treatment. We obtained a scaling factor of 1.46{plus minus}0.005 and 1.39{plus minus}0.005 for the mouse and rat data, respectively, in agreement with the published data.

The scaling factor results obtained with the help of total fluorescence intensity of PSD95 has been included in the third paragraph of the ‘Multiplicative scaling of scaffolding molecules during homeostatic scaling in rodents’ sub-section of the ‘Results’ section in the manuscript.

8) DISTANCE FROM SOMA

The authors never mention how far analyzed synapses were from the soma - but they should. It is important to know how scaling depends on dendritic distance - and whether that affects scaling. Given the kind of images the authors must possess, they should address this issue and include relevant images with this analysis.

Our analysis of scaling factors in figure 1 were performed on the proximal dendrite at a distance of 35-45 μm from the cell body. To examine how scaling depends on dendritic distance, we subsequently performed additional quantification on immunolabelled samples for GluA2 and Shank2 on DIV 16 rat hippocampal neurons which was treated for 48 hours with TTX. We used confocal microscopy to obtain tile scans of size 270x270 μm of individual cells. We selected 33X33 μm square regions with proximal (within ∼66 μm of the cell body) and distal (∼132 μm from the cell body) dendrites and performed our analysis on the selected puncta (Response to reviewers Figure 5). We observed that the distal dendrites scaled 5.94 - 7.12% more than the proximal dendrites.

Response to reviewers Figure 5: ‘Multiplicative scaling in proximal and distal synapses’

(A,B&C) 14DIV rat hippocampal neurons were treated with 2μM TTX for 48 hours, fixed and immunocytochemistry was performed for surface GluA2 (A) and Shank2 (B). A tile scan image of individual cells were taken with the cell body at the center, such that a total image size of 270X270 μm was acquired. (C) Shank2 (green) and GluA2 (magenta) tile scans were overlaid to indicate synapses in white. 2 concentric circular regions, one with a radius of 132μm and one with a radius of 66μm were drawn around the cell body. (A1-C1 & A2-C2) Square regions of 33X33μm were drawn within the first (1) and after the last circle (2) to differentiate between proximal (A1-C1) and distal dendrites (A2-C2). Scale bars for A,B&C indicate 50μm and for A1-C1 and A2-C2 indicate 8μm.

The microscopy and image analysis involved in acquiring and analysing the images for studying proximal and distal synapses has been included in the ‘Confocal Microscopy’ and ‘Image Analysis’ sub-section respectively under the ‘Materials and Methods’ section of the manuscript. The results relevant to the differential scaling observed between proximal and distal synapses has been included in the second paragraph under the ‘Differential scaling of global vs surface pools of GluA2 subunits of AMPA receptors at excitatory synapses’ sub-section of the ‘Results’ section.

9) SHANK2

The authors use the postsynaptic density protein Shank2 in their study, but never explain why.

Shank2 is a scaffolding protein present in the postsynaptic density of excitatory synapses which interacts with Homer and SAPAP which in turn interacts with PSD95. Shank protein has been shown to be a major component of the postsynaptic density of excitatory synapses (Brandstatter et al., 2004; Sheng and Hoogenraad, 2007; Chua et al., 2010). Shank2 has also been implicated in Autism Spectrum Disorders (ASD) (Berkel et al., 2010; Kim et al., 2018). We have also observed that the scaling between Shank2 and PSD95 at synapses were comparable. Additionally, antibodies against Shank2 were available from a Guinea pig host which was more suitable in combination with several AMPA receptor subunit antibodies, which were of key interest to the paper. Thus we have used Shank2 as a postsynaptic marker in our study. We have included our rationale for using Shank2 as the postsynaptic marker in our study, in the third paragraph under the ‘Multiplicative scaling of scaffolding molecules during homeostatic scaling in rodents’ sub-section of the ‘Results’ section in the manuscript.

10) AVERAGE INTENSITY

On the histograms, what is presently called “average intensity” might better be called “cluster intensity” with some indication that these are arbitrary units.

The term ‘cluster intensity’ was confused with integrated and average intensity among our peers, who had helped by looking through the readability and comprehension of our paper. Therefore, we reverted to ‘average intensity’ as it was discussed in the original manuscript.

References

Berkel S, Marshall CR, Weiss B, Howe J, Roeth R, Moog U, Endris V, Roberts W, Szatmari P, Pinto D, Bonin M, Riess A, Engels H, Sprengel R, Scherer SW, Rappold GA (2010) Mutations in the SHANK2 synaptic scaffolding gene in autism spectrum disorder and mental retardation. Nat Genet 42:489-491.

Brandstatter JH, Dick O, Boeckers TM (2004) The postsynaptic scaffold proteins ProSAP1/Shank2 and Homer1 are associated with glutamate receptor complexes at rat retinal synapses. J Comp Neurol 475:551-563.

Chua JJ, Kindler S, Boyken J, Jahn R (2010) The architecture of an excitatory synapse. J Cell Sci 123:819-823.

Engmann S, Cousineau D (2011) Comparing distributions: the two-sample Anderson-Darling test as an alternative to the Kolmogorov-Smirnov test. Journal of Applied Quantitative Methods 6:1-17.

Gainey MA, Hurvitz-Wolff JR, Lambo ME, Turrigiano GG (2009) Synaptic scaling requires the GluR2 subunit of the AMPA receptor. J Neurosci 29:6479-6489.

Han EB, Stevens CF (2009) Development regulates a switch between post- and presynaptic strengthening in response to activity deprivation. Proc Natl Acad Sci U S A 106:10817-10822.

Ibata K, Sun Q, Turrigiano GG (2008) Rapid synaptic scaling induced by changes in postsynaptic firing. Neuron 57:819-826.

Kim J, Tsien RW, Alger BE (2012) An improved test for detecting multiplicative homeostatic synaptic scaling. PLoS One 7:e37364.

Kim R, Kim J, Chung C, Ha S, Lee S, Lee E, Yoo YE, Kim W, Shin W, Kim E (2018) Cell-Type-Specific Shank2 Deletion in Mice Leads to Differential Synaptic and Behavioral Phenotypes. J Neurosci 38:4076-4092.

Scholz F, Stephens M (1987) K-Sample Anderson-Darling Tests of Fit, for Continuous and Discrete Cases. Journal of The American Statistical Association - J AMER STATIST ASSN 82.

Sheng M, Hoogenraad CC (2007) The postsynaptic architecture of excitatory synapses: a more quantitative view. Annu Rev Biochem 76:823-847.

Sun Q, Turrigiano GG (2011) PSD-95 and PSD-93 play critical but distinct roles in synaptic scaling up and down. J Neurosci 31:6800-6808.

Watt AJ, van Rossum MC, MacLeod KM, Nelson SB, Turrigiano GG (2000) Activity coregulates quantal AMPA and NMDA currents at neocortical synapses. Neuron 26:659-670.
